# Influence of CeO_2_ Nanoparticle Morphology on the Electrocatalytic Activity of Palladium toward the Formate Electrooxidation Reaction

**DOI:** 10.1021/acsomega.5c04822

**Published:** 2025-08-15

**Authors:** Aila O. Santos, Giulia K. Silva, Hozana S. C. Oliveira, Noemi R. C Huaman, André V. H. Soares, Odivaldo C. Alves, Júlio César M. Silva

**Affiliations:** † Departamento de Físico-Química, Instituto de Química, 28110 Universidade Federal Fluminense, Campus Valonguinho, 24020-141 Niterói, RJ, Brasil; ‡ 74350 Centro Brasileiro de Pesquisas Físicas, Urca, 22290-180 Rio de Janeiro, RJ, Brasil; § Departamento de Engenharia Química e de Petróleo, Universidade Federal Fluminense, Campus da Praia Vermelha, 24210-240 Niterói, RJ, Brasil

## Abstract

This study investigates the electrocatalytic activity of palladium (Pd) nanocatalysts combined with nanoparticles (NPs) of cerium oxide (CeO_2_) polyhedra (Pd/CeO_2_/C poly) and with morphologies of cube (Pd/CeO_2_/C NC), hexagonal sheet (Pd/CeO_2_/C NS), and nanorod (Pd/CeO_2_/C NR) for the formate electrooxidation reaction (FER) in an alkaline medium, a key process in direct formate fuel cells (DFFCs). X-ray diffraction (XRD) patterns indicate that the CeO_2_ NP dislocation density follows the decreasing order of Pd/CeO_2_/C NR > Pd/CeO_2_/C NS > Pd/CeO_2_/C NC > Pd/CeO_2_/C poly. This order corresponds to the Pd^0^ concentration observed in X-ray photoelectron spectroscopy (XPS) data. Pd/CeO_2_/C NR showed CeO_2_ NPs with compressive microstrain values, while the other materials presented tensile microstrain. Pd/CeO_2_/C NR showed a higher amount of oxygen vacancies in XPS analysis. Transmission electron microscopy (TEM) micrographs showed Pd NPs surrounding and in close contact with CeO_2_ NPs. The average Pd NPS size was 4–10 nm, while CeO_2_ NC, CeO_2_ NS, and CeO_2_ poly showed 30, 15, and 50 nm, respectively. The length and width of CeO_2_ NR were 100 and 10 nm, respectively. Cyclic voltammetry (CV) demonstrates that Pd/CeO_2_/C NR exhibits higher catalytic activity toward FER than the other materials studied (Pd/C, Pd/CeO_2_/C NC, Pd/CeO_2_/C NS, Pd/CeO_2_/C poly). Chronoamperometric analysis (CA) shows that the current density from FER on Pd/CeO_2_/C NR at the end of the experiment was 2.65 times higher than on Pd/C, while for Pd/CeO_2_/C NC and Pd/CeO_2_/C NS it was 1.45 and 1.13 times higher than on Pd/C. The enhanced catalytic activity of Pd/CeO_2_/C NR results from the interaction between palladium and ceria, involving microstrain, oxygen vacancies, and metal–support interaction, facilitating polar adsorbate adsorption. This makes Pd/CeO_2_/C NR a promising catalyst for direct formate fuel cell applications.

## Graphical abstract

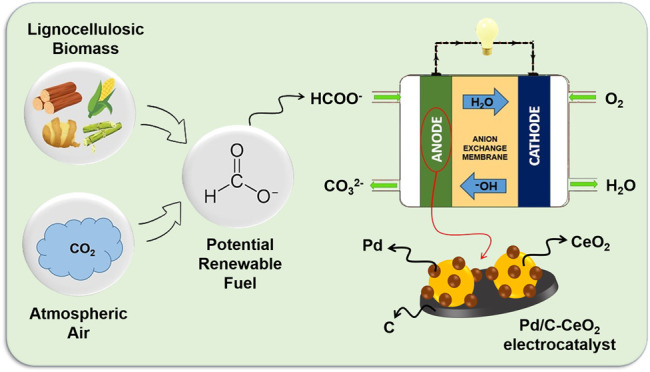

## 1 Introduction

Modern society has been extremely dependent on electricity since the advent of the Industrial Revolution,^1^ and with the world’s population growth, the demand for electricity in our society is increasing.^2^ Fossil fuels are the most used source in the energy matrix, resulting in a greater amount of greenhouse gases released into the atmosphere, which aggravates atmospheric pollution and the greenhouse effect.^3^ Moreover, beyond concerns about the environmental pollution generated during the burning of fossil fuels, the depletion of their energy reserves is another alarming factor, since these are not renewable, making dependence on these energy sources a critical global issue. Therefore, it is imperative to deploy technologies for electricity generation that are highly efficient and produce minimal pollutant emissions.^4^

In this context, the direct formate fuel cell (DFFC) is a promising device that enables the conversion of chemical energy from formate oxidation into electrical energy with high efficiency,^5^ resulting in low CO_2_ emissions.^6^ Bartrom and Haan^7^ reported that the overall theoretical energy of a fuel cell fueled by formate is 0.31 and 0.24 V higher than that of a fuel cell fueled by ethanol and methanol, respectively. The overall theoretical potential of the cell fueled by formate is reported to be 1.45 V,^7,8^ which is also 0.05 V higher than that of a cell fueled by formic acid.^6^

Formate is a sustainable fuel because it is nontoxic, noncorrosive, and nonflammable, which prevents storage and transportation accidents, and promotes low CO_2_ emissions.^6^ Additionally, it is easily handled, stored, transported, and can be readily converted into a liquid fuel due to its high solubility in water.^8,9^ Furthermore, formate is cost-effective^8^ and can be produced through electrochemical reduction of CO_2_ ^3,10–12^ and hydrolysis oxidation of lignocellulosic biomass.^13–15^ Formate synthesis can also be achieved via CO fixation, a synthesis gas released in various industrial exhausts such as steelworks, and is produced in biomass gasification and from residues like sewage sludge and municipal waste.^16^ However, electrooxidation of carbon-based fuels faces challenges due to poisoning by CO, an intermediate that adsorbs on the material surface, leading to catalyst deactivation and decreasing reaction kinetics and catalytic activity.^17^ Various strategies are adopted to enhance reaction kinetics and diminish poisoning effects on catalyst surfaces.^17^ Among these, nanostructured materials,^18^ semiconductor oxides,^19^ and morphological manipulation of nanoparticles^20,21^ are noteworthy.

Palladium-based nanocatalysts are extensively used in DFFCs due to palladium’s low affinity with CO, facilitating its oxidation and decreasing catalyst poisoning.^22^ Nanostructured metallic oxides are also widely studied for their ability to enhance catalytic activity and CO poisoning tolerance of catalyst active phases,^23,24^ attributed to their electronic and bifunctional effects at catalytic sites.^24,25^ Cerium IV oxide (ceria) is highly reactive among rare earth metal oxides, capable of storing and releasing oxygen species through reversible interconversion between Ce^4+^ and Ce^3+^ ions.^26,27^

Different morphologies of ceria nanoparticles expose distinct crystalline planes, influencing oxide physicochemical properties.^25^ CeO_2_ nanocubes preferentially expose the (100) plane,^28–32^ nanorods expose the (100) and (110) planes,^28–31,33^ and hexagonal nanosheets expose the (110) plane.^33–35^ The most thermodynamically stable surfaces for CeO_2_ are (111), (110), and (100), with surface energy and reactivity following the order (100) > (110) > (111).^25,33^ These exposed planes play a crucial role in improving the catalytic activity of ceria-based materials, as they directly affect surface energy, oxygen vacancy formation energy, and the availability of active sites. For instance, the highly reactive (100) and (110) planes facilitate oxygen mobility and redox cycling between Ce^4+^ and Ce^3+^, which are essential for oxidation–reduction reactions.^36,37^ As a result, tailoring ceria morphology to control the dominant exposed facets enables the rational design of catalysts with enhanced activity and selectivity for specific reactions.^36,37^

The design of high-performance electrocatalysts has increasingly focused on strategies that tune the electronic structure, optimize interfacial interactions, and expose catalytically active sites.^38–41^ Recent studies have demonstrated that combining multiple structural engineering approaches—such as heteroatom doping, interface modification, and control over anion coordination—can enhance charge redistribution and improve electron transfer at the active surface.^38,39^ In particular, catalysts derived from MOF-based frameworks or containing hybrid architectures benefit from *in situ* surface reconstruction under alkaline conditions, leading to new active phases and improved electrochemical behavior.^39–41^ The presence of compositional gradients, layered or hollow morphologies, and internal electric fields at heterojunctions has also been shown to modulate the electronic environment of transition metal centers, thereby facilitating key steps in catalytic reactions.^40,41^ Although these advances have been largely applied to water splitting reactions, the underlying concepts—namely, electronic tuning, defect formation, and interfacial engineering—are directly relevant to systems based on Pd nanoparticles supported on CeO_2_. In such systems, the metal–support interaction (MSI) and the presence of oxygen vacancies and morphological control play a crucial role in governing the catalytic performance toward formate electrooxidation.

In this study, Pd/C and Pd/CeO_2_/C catalysts with different morphologies of CeO_2_ nanoparticles NC, NS, and NR were synthesized and used as electrocatalysts for formate electrooxidation in alkaline media. CeO_2_ nanoparticles were synthesized via the hydrothermal method, and palladium nanoparticles were synthesized using chemical reduction with NaBH_4_. The catalysts were characterized using TEM, EDS, XRD, and XPS.

## 2 Materials and Methods

### 2.1 Chemicals and Materials

Ultrapure water (18 MΩ cm), sodium hydroxide (NaOH > 99%, Impex), ethanol (CH_3_CH_2_OH > 99.50%, Proquímicos), cerium­(III) nitrate hexahydrate (Ce­(NO_3_)_3_·6H_2_O > 99%, Sigma-Aldrich), ammonium hydroxide solution (NH_4_OH > 28%, ACS reagent), polyhedral cerium­(IV) oxide (CeO_2_ > 99%, Sigma-Aldrich), Nafion (5%, Sigma-Aldrich), isopropyl alcohol ((CH_3_)_2_CHOH > 99.50%, Sigma-Aldrich), sodium formate (HCOONa > 99.50%, Sigma-Aldrich), palladium­(II) nitrate dihydrate (Pd­(NO_3_)_2_·2H_2_O, 40% Pd, Sigma-Aldrich), Vulcan carbon (X72R—Cabot Corporation), and sodium borohydride (NaBH_4_ > 99%, Sigma-Aldrich) were used.

### 2.2 CeO_2_ Nanoparticle Synthesis

Ceria nanoparticle synthesis was performed by a hydrothermal method using Ce­(NO_3_)_3_·6H_2_O as the cerium precursor. A dispersion containing Ce­(NO_3_)_3_·6H_2_O and the mineralizer agent was magnetically stirred for 20 min at room temperature and then transferred to a Teflon-lined stainless steel autoclave, which was placed in an oven at a suitable time and temperature.^34,42^ To synthesize CeO_2_ NR, the method reported by Trenque et al.^42^ was adapted. The CeO_2_ NC and NS were synthesized according to the methods reported by Trenque et al.^42^ and Liu et al.,^34^ respectively. Table 1 presents the parameters used in the synthesis of each CeO_2_ morphology. The total volume refers to the volume resulting from the mixture of all reactants. After the hydrothermal synthesis process, the autoclave was naturally cooled down to room temperature. The product was centrifuged at 10,000 rpm and washed with water and ethanol two and three times, respectively, and then dried at 85 °C for 24 h.^42^

**Table 1:** Synthetic Parameters of Ceria Nanoparticle Preparation

	CeO_2_ NC	CeO_2_ NS	CeO_2_ NR
Ce(NO_3_)_3_·6H_2_O	0.05 mol L^–1^	0.03 mol L^–1^	0.086 mol L^–1^
mineralizer	NaOH 6.00 mol L^–1^	NH_4_OH 0.016 mol L^–1^	NaOH 2.00 mol L^–1^
total volume	53.45 mL	90.00 mL	31.00 mL
temperature	180 °C	220 °C	100 °C
time	24 h	24 h	10 h

### 2.3 Pd Nanoparticle Synthesis and Preparation of Pd/C and Pd/CeO_2_/C

The synthesis of palladium polycrystalline nanoparticles supported on carbon (C) or ceria-carbon (CeO_2_/C) was carried out via the chemical reduction method using sodium borohydride.^43^ The metallic precursor used was Pd­(NO_3_)_2_·2H_2_O. For the synthesis of the Pd/C electrocatalyst, a dispersion containing a mixture of 25 mL of isopropyl alcohol and 25 mL of water with 135 mg of Carbon Vulcan was homogenized using ultrasound for 5 min. Subsequently, 37.56 mg of Pd­(NO_3_)_2_·2H_2_O was added to the carbon dispersion, and the mixture was magnetically stirred for 5 min. A reductive solution was prepared by adding 26.90 mg of NaBH_4_ to 10 mL of a 0.1 mol L^–1^ KOH solution (pH = 14) and kept cold to stabilize NaBH_4_. The reductive solution was then added in a single portion to the dispersion containing the palladium precursor (1:5 Pd^2+^:NaBH_4_) and carbon, under vigorous stirring at room temperature, and maintained for 15 min.^43,44^ Finally, the dispersion was centrifuged at 10,000 rpm to collect the precipitate. The material was washed with ethanol twice and dried at 85 °C for 24 h. The intended palladium metal loading and carbon content were 10% Pd and 90% C, respectively.^19,45,46^

For the synthesis of the Pd/CeO_2_/C electrocatalysts, a mixture of 25 mL of isopropyl alcohol, 25 mL of water, and 67.50 mg of ceria nanoparticles was prepared. Then, 37.56 mg of the palladium precursor was added to the solution, and the mixture was magnetically stirred for 5 min. A reductive solution was prepared by dissolving 26.90 mg of NaBH_4_ in 10 mL of 0.1 mol L^–1^ KOH solution, and kept cold. This solution was subsequently added in one portion to the dispersion containing the palladium precursor (1:5 Pd^2+^:NaBH_4_) and ceria, under vigorous stirring at room temperature and kept under these conditions for 15 min.^43,44^ The dispersion was centrifuged at 10,000 rpm to remove the supernatant containing reaction byproducts. A mixture containing 50 mL of ultrapure water and 67.50 mg of Carbon Vulcan was then homogenized using ultrasound for 5 min. The Pd/CeO_2_ nanoparticles were added to the carbon dispersion and left under vigorous stirring at room temperature for 6 h.^21^ Finally, the mixture was centrifuged at 10,000 rpm, and the precipitate was collected, washed with ethanol twice, and dried at 85 °C for 24 h. The palladium metal loading and the planned proportions of ceria and carbon were 10% Pd,^19,45,46^ 45% CeO_2_, and 45% C.^19,47^

### 2.4 Physicochemical Characterizations

X-ray diffraction was carried out using an X’Pert Pro (Panalytical) PW3042 diffractometer with Cu Kα radiation equal to 0.15406 nm (40 kV, 40 mA) and an X-Celerator solid-state detector. The diffraction patterns were obtained in a 2θ interval between 10 and 90° and at a scan rate of 0.025 °s^–1^ at room temperature and atmospheric pressure. The Inorganic Crystal Structure Database (ICSD) was used to identify the crystalline structures. The ICSD reference numbers for CeO_2_, palladium, and graphite carbon were #072155 (PDF #01-081-0792), #76148 (PDF #01-089-4897), and #31170 (PDF #01-07-51621), respectively. The Rietveld method was employed to refine all complete diffraction profiles. A Pseudo-Voigt function was adopted to model the peak shape.^48–51^ The instrumental broadening was subtracted using a silicon standard.^52^ The background of the diffraction profile was fitted using a polynomial function.^51^ The refinement of the diffraction patterns was performed by fixing the atomic positions of the metals and setting their occupancies to 100%. The oxygen atom occupancy was adjusted based on XPS results for oxygen vacancies, ensuring consistency between the DRX and XPS analyses.^53^ Additionally, scale factor, background, sample displacement, zero shift displacement, Caglioti U, V, and W parameters, peak shape (Form 1), peak asymmetry parameters, and lattice parameter ***a*** were also refined.^54^

The size and morphology, and dispersion of the NPs were analyzed via electron microscopy in transmission (TEM) and scanning modes STEM using a JEOL 2100F microscope operating at 200 kV, equipped with a CMOS camera ONE VIEW for TEM imaging and energy-dispersive X-ray spectroscopy (EDS) for NP dispersion assessment. Elemental composition was evaluated by scanning electron microscopy (SEM) using a Hitachi FlexSem 1000II at 15 kV with EDS. SEM-EDS analyses encompassed the entire sample holder to ensure representative quantification, whereas TEM-EDS focused on specific regions to assess localized elemental dispersion. The element carbon was not included in these analyses due to equipment limitations related to its detection. For both techniques, a suspension of the material in isopropyl alcohol was prepared.^55,56^

X-ray photoelectron spectroscopy was performed to analyze the composition and chemical state of atoms on the surface of the catalyst sample using an ESCALAB 250XI (Thermo Fisher Scientific) with monochromatic Al Kα X-ray radiation (1486.68 eV) under ultrahigh vacuum conditions at room temperature. The Shirley method was adopted to subtract the background spectra,^57,58^ calibrated with the C 1s photoemission peak.^59^ The deconvolution and the integrated area of the curves were fitted by a Gaussian distribution function.^57,60^ Peaks positions were attributed according to the literature.^57,61–63^

### 2.5 Catalytic Dispersion, Preparation, and Electrochemical Measurements

The catalytic dispersion was prepared using 6.00 mg of catalyst, 900 μL of deionized water, 100 μL of isopropyl alcohol, and 20 μL of Nafion. The dispersion was homogenized under ultrasound for 10 min. Then, 8 μL was deposited on the working electrode, dried at 65 °C for 10 min, and inserted into the three-electrode electrochemical cell.^55^ The working electrode, counter electrode, and reference electrode were glassy carbon, platinum, and Hg|HgO electrodes, respectively.

Electrochemical characterization in N_2_-saturated 1.0 mol L^–1^ NaOH solution was performed by Cyclic Voltammetry measurements, which were carried out from −0.85 to 0.10 V with a scan rate of 50 mV s^–1^ for 10 cycles.^22^ The electrocatalytic activity of the materials was investigated by CV experiments in 1.0 mol L^–1^ NaOH with 1.0 mol L^–1^ HCOONa. For this purpose, 5 cycles with a scan rate of 20 mV s^–1^ from −0.85 to 0.10 V were collected.^64,65^ Chronoamperometric analysis at −0.55 V for 1800 s in 1.0 mol L^–1^ NaOH with 1.0 mol L^–1^ HCOONa was carried out to obtain information about the electrocatalytic stability.^22^ The current produced from the formate electrooxidation reaction was normalized per electrochemical surface area (ECSA) according to eq 1.

1 $$\mathrm{ECSA}=\frac{Q}{405\times m_{\mathrm{Pd}}}$$

Where *Q* is the amount of charge exchanged during the Pd–O monolayer reduction on the Pd surface **μ** ***C***; 405 μC cm^–2^ is the charge required to reduce the oxide monolayer; and ***m***_**Pd**_ is the palladium loading in the electrode.^22,65–68^

## 3 Results and Discussion

### 3.1 Physical Chemical Characterization

Figure 1 presents the XRD patterns of the catalyst materials refined using the Rietveld method. The characteristic peaks of the fluorite-type face-centered cubic structure (space group *Fm*3̅*m*) of CeO_2_ at about 29, 33, 47, 56, 59, 69, 77, 80, and 89°, which correspond to the crystalline planes (111), (200), (220), (311), (222), (400), (331), (420), and (422), respectively, are marked in the Pd/CeO_2_/C NC diffractogram.^69–71^ A diffraction peak at 2θ = 25°, related to the reflection plane (002) of the graphite in Carbon Vulcan^24,72–74^ exhibits low intensity in Pd/CeO_2_/C materials.

**Figure 1:**
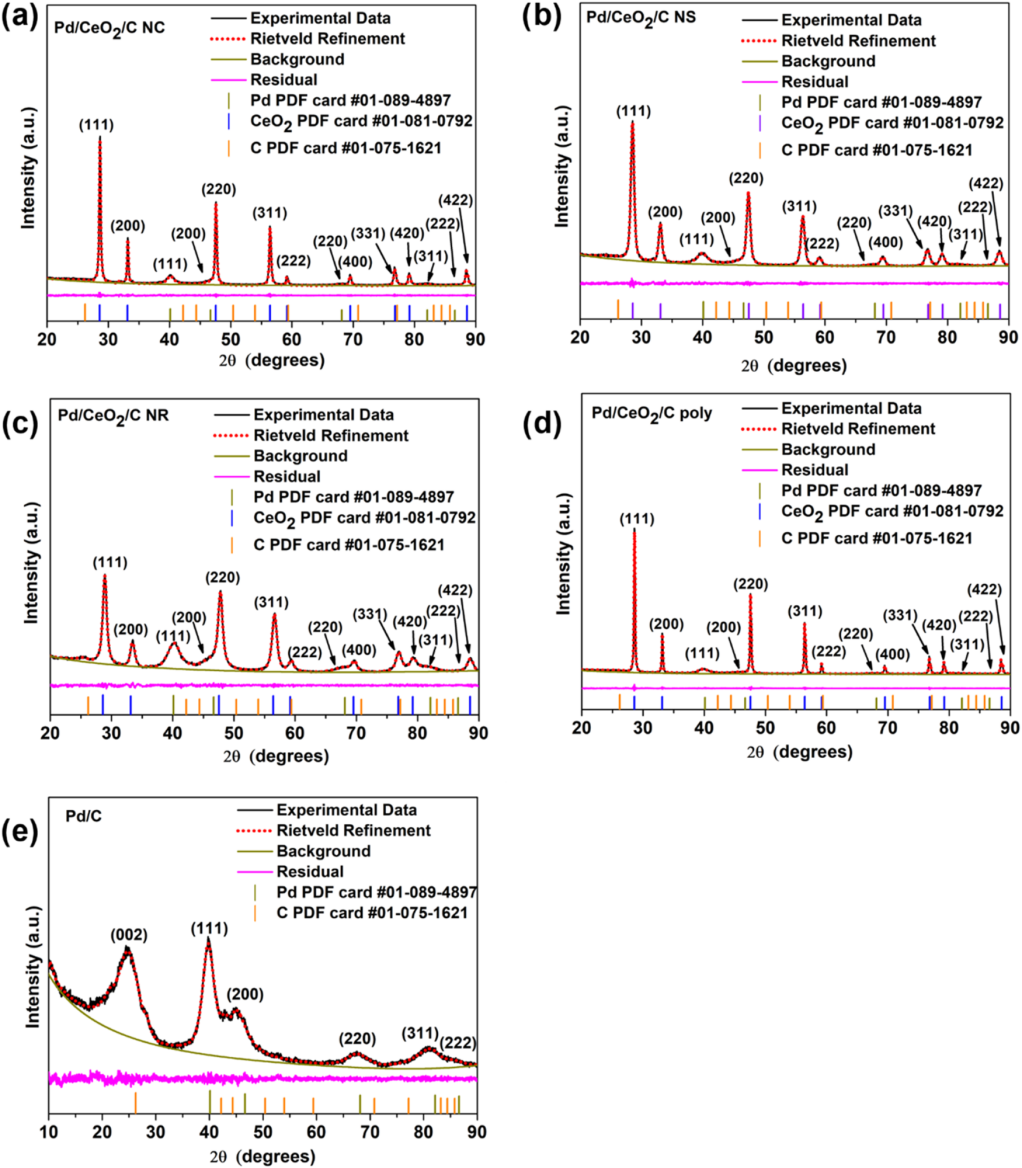
XRD patterns and Rietveld Refinement of (a) Pd/CeO_2_/C NC, (b) Pd/CeO_2_/C NS, (c) Pd/CeO_2_/C NR, (d) Pd/CeO_2_/C poly, and (e) Pd/C.

The characteristic peaks of the face-centered cubic (space group *Fm*3̅*m*) of Pd at about 40, 45, 68, 81, and 87° related to the crystalline planes (111), (200), (220), (311), and (222) respectively, are marked in the diffraction patterns of Pd/C.^47^ The diffraction peak of Pd with the highest intensity, located at 40°, is observed in all Pd/CeO_2_/C materials. However, the other Pd peaks appear with low intensity, possibly due to the higher crystallinity of the ceria compared to the palladium nanoparticles. The peak positions of Pd and CeO_2_ are consistent with the crystallographic reference cards used in the analysis, confirming a good agreement between the experimental data and the reference patterns. Sharper peaks observed for the polyhedral and cubic ceria nanoparticles suggest higher crystallinity and larger crystallite sizes. In contrast, broader peaks for the hexagonal sheet and nanorod morphologies indicate lower crystallinity and smaller crystallite sizes.

The goodness-of-fit ***GOF*** indices of the Rietveld refinement, calculated using eq 2, are defined as the ratio of the squared weighted profile *R* factor ***R***_***wp***_^***2***^ to the squared experimental *R* factor ***R***_***exp***_^***2***^, which provides a measure of the agreement between the experimental data and the refined profile.^54,75^ Table 2 presents the Rietveld refinement indices of agreement and demonstrates a good correspondence between the model and the experimental data, indicating a suitable fit.^54,75^ presents refined XRD patterns using the Rietveld refinement method, highlighting the comparison between experimental and calculated profiles.

2 $$GOF\;\left(\chi^{2}\right)=\frac{R_{wp}^{2}}{R_{\exp}^{2}}$$

**Table 2:** Rietveld Refinement Parameters Obtained from XRD Pattern of Catalyst Materials

	*R*_exp_	*R*_wp_	GOF
Pd/CeO_2_/C NC	4.48	4.78	1.07
Pd/CeO_2_/C NS	4.31	4.66	1.08
Pd/CeO_2_/C NR	3.75	3.95	1.05
Pd/CeO_2_/C poly	4.26	4.74	1.11
Pd/C	3.48	3.58	1.03

Table 3 summarizes the lattice parameter ***a*** with the associated deviation and the relative percentual quantities of palladium and ceria NPs determined via Rietveld refinement of the diffraction patterns. For the Pd/C catalyst, the graphite carbon crystallographic card was employed to differentiate the contributions of carbon and palladium, given the low crystallinity of palladium. When palladium is present solely with carbon, the carbon contribution to X-ray diffraction becomes significant. In contrast, for materials containing ceria, this contribution is negligible due to the high crystallinity of CeO_2_. According to the results in Table 3, CeO_2_ with controlled morphology showed an increase in the lattice parameter, while polyhedral ceria exhibited a decrease. This increase in lattice parameters suggests the presence of defects in the structure, such as oxygen vacancies, which lead to structural relaxation by weakening the Ce–O bonds at the surface.^76–78^ This weakening of the Ce–O bonds may influence the material’s physicochemical properties and alter its reactivity and magnetic properties.^76–78^ The lattice parameter values for the ceria nanoparticles in the electrocatalysts followed the decreasing order: Pd/CeO_2_/C NR, Pd/CeO_2_/C NC, Pd/CeO_2_/C NS, Pd/CeO_2_/C poly.

**Table 3:** Lattice Parameters and Semiquantitative Phase Analysis Derived from Rietveld Refinement of the XRD Patterns for Pd/C and Pd/CeO_2_/C

	CeO_2_		Pd	
	*a* (Å)	*C*_CeO_2__ (%)	*a* (Å)	*C*_Pd_ (%)
Pd/CeO_2_/C NC	5.4148 ± 0.0001	82	3.9013 ± 0.0001	18
Pd/CeO_2_/C NS	5.4130 ± 0.0001	75	3.9110 ± 0.0005	25
Pd/CeO_2_/C NR	5.4272 ± 0.0002	81	3.9277 ± 0.0008	19
Pd/CeO_2_/C poly	5.4116 ± 0.0001	79	3.9189 ± 0.0006	21
Pd/C	x	x	3.9366 ± 0.0019	10

A contrasting result was reported in the literature, where palladium addition to CeO_2_ NR, CeO_2_ NC, and CeO_2_ poly NPs resulted in an increase in the lattice parameter.^79^ This discrepancy can be attributed to the preparation method, which involved vacuum drying at 80 °C for 16 h, followed by calcination at 500 °C for 4 h, aimed at removing oxygenated species adsorbed on the ceria NPs surface and inducing crystal defects such as oxygen vacancies.^79^ Additionally, it was observed that among the ceria-containing materials, Pd/CeO_2_/C NS exhibited the highest palladium content, while Pd/CeO_2_/C NC showed the lowest. The palladium content in the samples followed the decreasing order: Pd/CeO_2_/C NS, Pd/CeO_2_/C poly, Pd/CeO_2_/C NR, Pd/CeO_2_/C NC. The addition of CeO_2_ in catalysts results in a decrease in the lattice parameter of palladium nanoparticles compared to Pd/C.

The analysis of X-ray diffraction peak broadening allows for the indirect determination of nanocrystal size and intrinsic strain.^80–84^ Theoretical mathematical models can be used to extract structural information from the XRD profiles.^85^ These models examine the broadening of the peaks in the diffractograms and determine the crystallographic parameters, with the Scherrer model and SSP plot being particularly significant.^80–84^ Both methods rely on linear regression to predict the desired parameters. The Scherrer method accounts solely for the effect of crystallite size on X-ray diffraction peak broadening, neglecting intrinsic strain caused by crystallographic defects such as point defects, dislocations, grain boundaries, phase junctions, and stacking faults.^80–84^ Equation 3 was used to estimate the crystallite size ***D*** using the Scherrer method, where ***λ*** represents the X-ray wavelength (λ = 0.15406 nm), ***β*** is the full width at half-maximum of the diffraction peak, ***θ*** is the Bragg diffraction angle, and ***k*** is the Scherrer constant (*k* = 0.9). The expression was linearly fitted, with ***1*** **/** ***β*** and ***cos*** **(** ***θ*** **)** as the ***X*** and ***Y*** variables, respectively. The slope corresponds to ***1*** **/** ***D***.^80–84^

3 $$\mathrm{co}s\left(\theta \right)=\frac{1}{\beta }.\frac{k\lambda }{D}$$

The Size-Strain Plot ***SSP*** method analyzes the peak profile, modeling the X-ray diffraction pattern as a combination of Lorentzian functions (for broadening due to size) and Gaussian functions (for broadening due to strain).^80–84,86^ The SSP method assumes that smaller angles are more effective in estimating size and strain in anisotropic crystals compared to larger angles. It is particularly suited for the favorable region of XRD analysis at lower angles. Equation 4 presents the expression used in the SSP method, where ***d***_***hkl***_ denotes the interplanar distance. This approach yields results with reduced errors by neglecting the contribution of high-angle diffraction peaks.^80–84,86^ Hence, eq 4 was applied for the linear fitting of the SSP model, where the variables ***X*** and ***Y*** correspond to **(** ***d***_***hkl***_^**2**^ ***βcos*** **(** ***θ*** **))** and **(** ***d***_***hkl***_ ***βcos*** **(** ***θ*** **))** ^**2**^, respectively. The slope is defined as $\frac{k\lambda }{D}$ , while the intercept is $\frac{\varepsilon^{2}}{4}$ .^80–84,86^

4 $$(d_{\mathrm{hkl}}\beta \mathrm{co}s{\left(\theta \right))}^{2}=\frac{k\lambda }{D}(d_{\mathrm{hkl}}^{2}\beta \mathrm{co}s\left(\theta \right))+\frac{\varepsilon^{2}}{4}$$

The dislocation density ***δ*** was estimated using eq 5 to analyze the presence of surface and linear crystal defects.^87–90^ Dislocation density refers to the total length of dislocation lines present in a unit volume of the material, providing insights into the defect structure and mechanical properties of the material.^91^ The mobility of dislocations, which refers to their capacity to move under an applied stress, is crucial for understanding the material’s deformation behavior. This mobility directly influences the material’s mechanical properties, particularly in nanoscale systems where defect dynamics play a significant role.^77,92^

5 $$\delta =\frac{1}{D^{2}}$$

Figures S1 and S2 present the linear fittings obtained using the Scherrer and SSP models for ceria and palladium nanoparticles, respectively. The Scherrer model evaluates peak broadening exclusively as a function of crystallite size, offering a simple approach to estimating particle dimensions. Conversely, the SSP model incorporates both crystallite size and microstrain effects, employing a combination of Lorentzian and Gaussian components to achieve a more detailed characterization of peak broadening. The higher *R* ^2^ values observed for the SSP model, in comparison to the Scherrer model, underline its enhanced precision and suitability for accurately assessing the structural features of the nanoparticles. Table 4 presents the results obtained from the Scherrer and SSP methods, applied to the Rietveld-refined XRD patterns of CeO_2_ NPs in the Pd/C-CeO_2_ samples. In contrast, Table 5 details the structural parameters of Pd NPs within the Pd/C and Pd/CeO_2_/C system. It emphasizes the impact of ceria morphology on the crystallite size, microstrain, and dislocation density of Pd NPs, enabling a comparative evaluation of their structural properties.

**Table 4:** Results from the Scherrer and SSP Methods Obtained from the Rietveld-Refined XRD Patterns for CeO_2_ NPs in the Pd/CeO_2_/C Samples

		Scherrer	SSP
Pd/CeO_2_/C NC	*D* (Å)	439	298
	ε (10^–6^)	X	6.69
	δ (10^–6^ Å^–2^)	5.19	11.3
Pd/CeO_2_/C NS	*D* (Å)	116	103
	ε (10^–6^)	X	7.11
	δ (10^–6^ Å^–2^)	74.3	94.2
Pd/CeO_2_/C NR	*D* (Å)	91.6	82.1
	ε (10^–6^)	X	–20.8
	δ (10^–6^ Å^–2^)	119	148
Pd/CeO_2_/C poly	*D* (Å)	900	502
	ε (10^–6^)	X	0.435
	δ (10^–6^ Å^–2^)	1.23	3.96

**Table 5:** Results from the Scherrer and SSP Methods Obtained from the Rietveld-Refined XRD Patterns for Pd NPs in the Pd/C and Pd/CeO_2_/C Samples

		Scherrer	SSP
Pd/CeO_2_/C NC	*D* (Å)	102	99.9
	ε (10^–3^)	X	6.96
	δ (10^–4^ Å^–2^)	0.954	1.00
Pd/CeO_2_/C NS	*D* (Å)	39.4	27.0
	ε (10^–3^)	X	2.64
	δ (10^–4^ Å^–2^)	6.44	13.8
Pd/CeO_2_/C NR	*D* (Å)	72.0	46.5
	ε (10^–3^)	X	5.08
	δ (10^–4^ Å^–2^)	1.93	4.62
Pd/CeO_2_/C poly	*D* (Å)	34.3	29.2
	ε (10^–3^)	X	5.72
	δ (10^–4^ Å^–2^)	8.50	11.7
Pd/C	*D* (Å)	49.1	32.7
	ε (10^–3^)	X	14.8
	δ (10^–4^ Å^–2^)	4.15	9.33

The analysis of the structural parameters of the Pd/CeO_2_/C samples, as summarized in Table 4, reveals distinct effects in lattice microstrain, crystallite size, and dislocation density across the different CeO_2_ morphologies. The crystallite size values derived from both the Scherrer and SSP models show discrepancies. The SSP model, which incorporates both crystallite size and microstrain, consistently provided smaller crystallite sizes, highlighting its greater sensitivity to lattice distortions.^80–84,86^ The crystallite size effects further emphasize the role of morphology, with Pd/CeO_2_/C poly exhibiting the largest crystallites, while Pd/CeO_2_/C NR showed the smallest.

Analyzing the microstrain values, it was observed that CeO_2_ NPs in Pd/CeO_2_/C NS exhibited the highest tensile microstrain (ε > **0**), followed by Pd/CeO_2_/C NC and Pd/CeO_2_/C poly, while Pd/CeO_2_/C NR showed compressive microstrain (ε < **0**).^76–78^ These results underscore the influence of NP morphology on lattice distortion, with tensile strain associated with Ce–O bond elongation and compressive strain to bond contraction.^76–78^ Such distortions significantly affect the material’s electronic structure and catalytic activity.^87^ Regarding dislocation density, Pd/CeO_2_/C NR exhibited the highest values by the SSP method, followed by Pd/CeO_2_/C NS, Pd/CeO_2_/C NC, and Pd/CeO_2_/C poly. The elevated dislocation density in Pd/CeO_2_/C NR suggests a higher concentration of crystal defects, indicative of a more stressed and deformed structure. In contrast, the lower dislocation density in Pd/CeO_2_/C poly indicates a more relaxed structure.^93^

The results in Table 5 indicate that the incorporation of ceria into the Pd/C system led to a more compressed lattice structure in Pd NPs compared to Pd/C, driven by a decrease in microstrain values.^76–78^ Notably, all Pd NPs exhibited tensile microstrain. Comparing the Pd NPs microstrain values, Pd/C exhibited the highest Pd NPs microstrain, followed by Pd/CeO_2_/C NC, P Pd/CeO_2_/C poly, Pd/CeO_2_/C NR, and Pd/CeO_2_/C NS. In terms of dislocation density, Pd NPs in Pd/CeO_2_/C NS presented the highest value, followed by Pd/CeO_2_/C poly, Pd/C, Pd/CeO_2_/C NR, and Pd/CeO_2_/C NC. The crystallite size analysis revealed that Pd NPs in Pd/CeO_2_/C NC had the largest size, whereas Pd/CeO_2_/C NS exhibited the smallest crystallites.

TEM micrographs of the palladium-based nanomaterials are presented in Figure 2. The larger images correspond to pure ceria materials, while the inset images depict the electrocatalysts. The micrographs reveal distinct ceria morphologies, including nanocubes (Figure 2a), hexagonal nanosheets (Figure 2b), nanorods (Figure 2c), and randomly shaped polyhedral structures (Figure 2d). The average ceria NPs sizes align with those reported in the literature for the applied synthesis methodologies.^34,42,94^ According to literature, CeO_2_ NC predominantly exposes the (100) plane,^28–31^ CeO_2_ NR exposes the (100) and (110) planes,^28–31^ CeO_2_ NS the (110) plane,^34,35^ and CeO_2_ poly the thermodynamically stable (111) plane.^93^ The lattice spacings for the (110), (100), and (111) planes are 0.19, 0.27, and 0.33 nm, respectively.^29,95^

**Figure 2:**
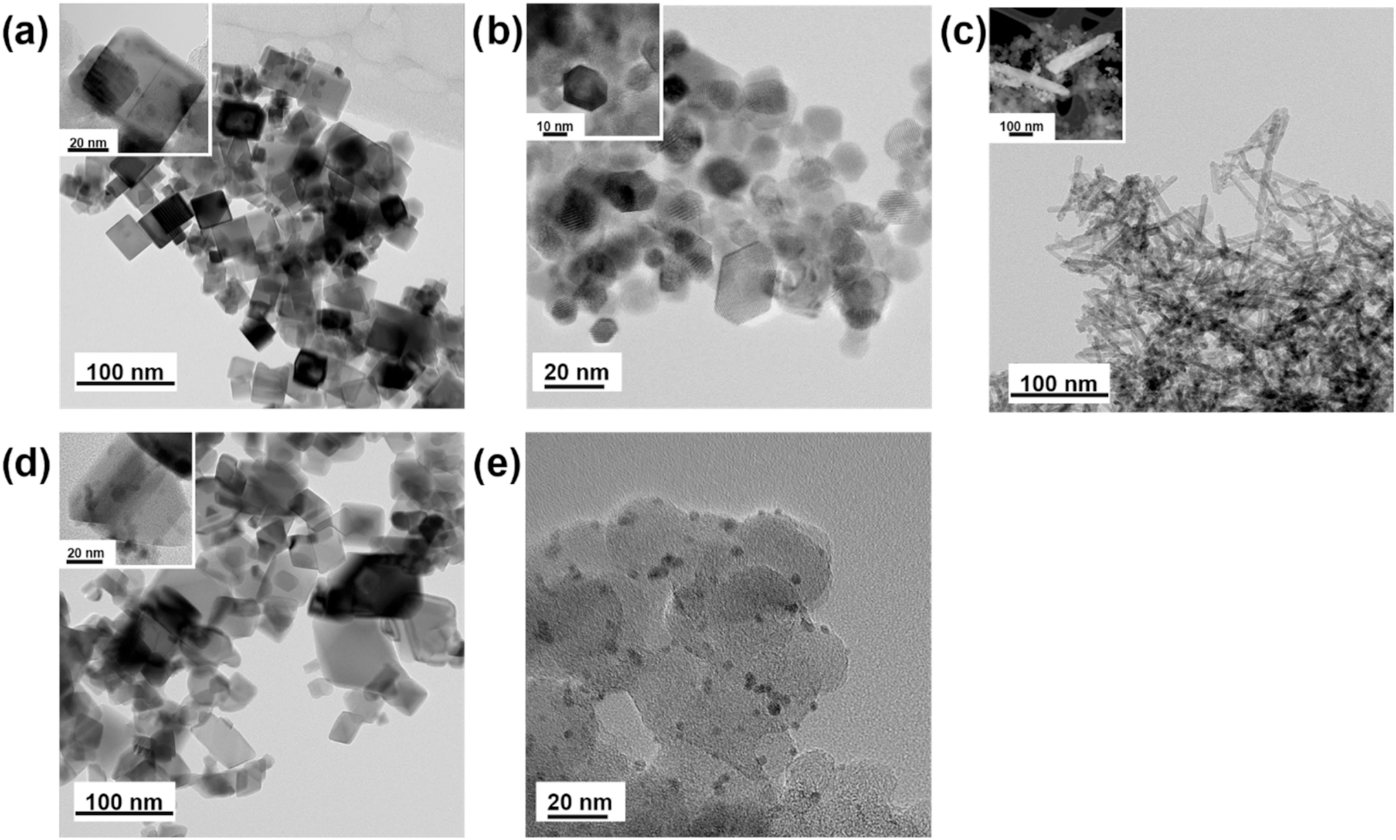
TEM and STEM micrographs of (a) Pd/CeO_2_/C NC, (b) Pd/CeO_2_/C NS, (c) Pd/CeO_2_/C NR, (d) Pd/CeO_2_/C poly, and (e) Pd/C.

All catalysts showed palladium nanoparticles with average sizes ranging from 4 to 10 nm. Pd/CeO_2_/C NC exhibited ceria NPs with an average size of 30 nm, Pd/CeO_2_/C NS showed ceria NPs averaging 15 nm, Pd/CeO_2_/C NR presented ceria NPs with 10 nm cross sections and 100 nm lengths, and Pd/CeO_2_/C poly featured ceria NPs averaging 50 nm. These dimensions are consistent with the literature.^43,44^ The NP sizes determined by TEM correspond to those derived from XRD (Tables 2 and 3), where crystallite sizes are comparable to the observed NP dimensions. For rod-shaped particles, the crystallite size matches the cross-sectional size, validating the reliability of the size estimates obtained through the Scherrer method, SSP model, and Rietveld refinement.

The STEM and the chemical mapping images obtained by TEM-EDS are presented in Figure 3, along with their respective elemental maps for the palladium-based nanomaterials. The presence of palladium NPs deposited on ceria and carbon NPs, as well as near ceria NPs, was evident. Table 6 presents the atomic ratio ***At%*** of the elements obtained from chemical mapping by SEM-EDS. The relative palladium content in the samples follows the order Pd/CeO_2_/C NS, Pd/CeO_2_/C poly, Pd/CeO_2_/C NR, and Pd/CeO_2_/C NC. The relative cerium content in Pd/CeO_2_/C follows the order Pd/CeO_2_/C NR, Pd/CeO_2_/C poly, Pd/CeO_2_/C NS, Pd/CeO_2_/C NC. The oxygen content followed the order Pd/CeO_2_/C NC, Pd/CeO_2_/C NS, Pd/CeO_2_/C poly, and Pd/CeO_2_/C NR.

**Figure 3:**
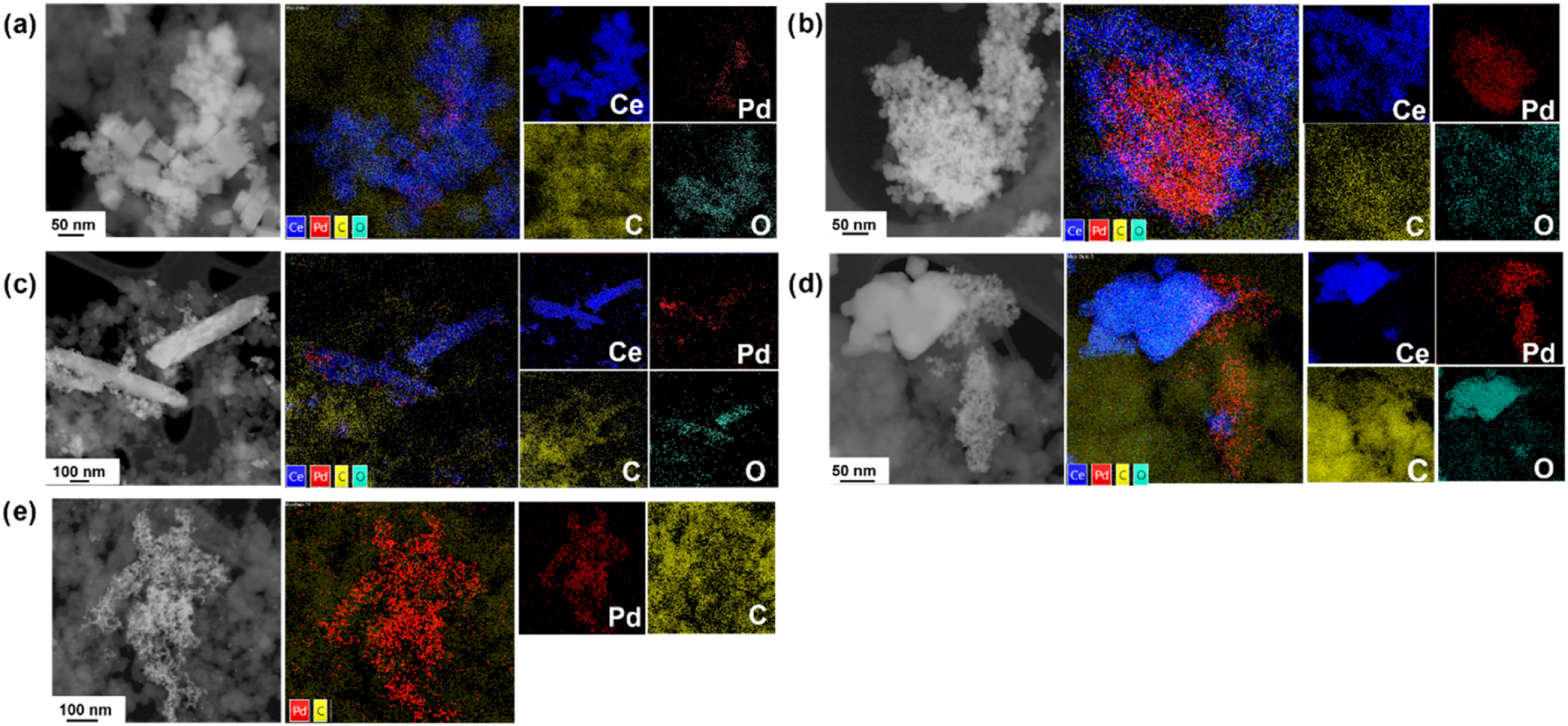
STEM micrographs and EDS mapping of (a) Pd/CeO_2_/C NC, (b) Pd/CeO_2_/C NS, (c) Pd/CeO_2_/C NR, (d) Pd/CeO_2_/C poly, and (e) Pd/C.

**Table 6:** Relative Amounts of Elements Present in Pd/C and Pd/CeO_2_/C Obtained by SEM-EDS

	at% Ce	at% O	at% Pd
Pd/CeO_2_/C NC	62.6	20.3	16.9
Pd/CeO_2_/C NS	62.7	14.7	22.6
Pd/CeO_2_/C NR	68.1	14.1	17.8
Pd/CeO_2_/C poly	64.9	14.6	20.5
Pd/C	X	16.9	83.1

Figure 4 presents the XPS survey spectra of the Pd/C and Pd/C-CeO_2_ samples, highlighting the characteristic electron emission peaks corresponding to Cerium (Ce 3d), Oxygen (O 1s), Carbon (C 1s), and Palladium (Pd 3d). The atomic ratios of Ce 3d, O 1s, C 1s, and Pd 3d are detailed in Table 7. Among the ceria-containing samples, Pd/CeO_2_/C NC exhibited the highest surface oxygen content, whereas Pd/CeO_2_/C poly presented the lowest. In terms of cerium content, Pd/CeO_2_/C NS showed the highest concentration, while Pd/CeO_2_/C poly exhibited the lowest, indicating a slight variation among the ceria-containing samples. Regarding palladium, Pd/CeO_2_/C NS demonstrated the highest concentration among the ceria-based materials, whereas Pd/CeO_2_/C NC exhibited the lowest.

**Figure 4:**
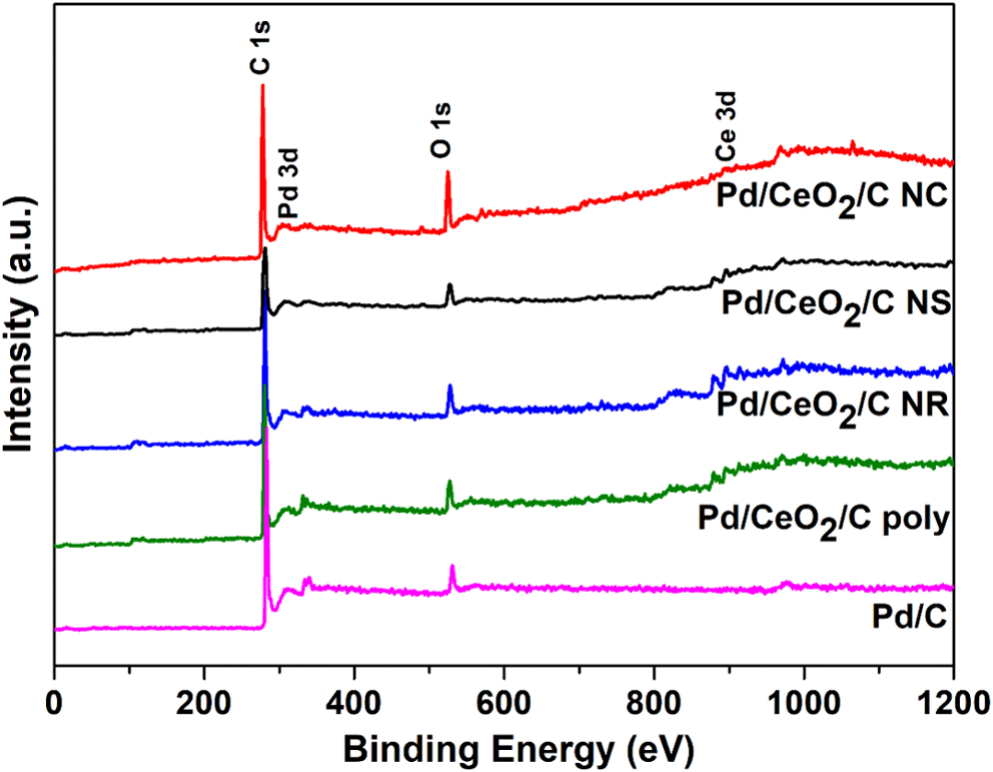
XPS survey spectra of Pd/C and Pd/CeO_2_/C nanomaterials.

**Table 7:** Atomic Ratios and Position of Ce 3d, O 1s, Pd 3d, and C 1s Obtained from the XPS Survey Spectra of Pd/C and Pd/CeO_2_/C

	At% Ce 3d	At% O 1s	At% Pd 3d	At% C 1s
Pd/CeO_2_/C NC	35.8	11.9	9.81	42.4
Pd/CeO_2_/C NS	38.5	7.78	9.92	43.8
Pd/CeO_2_/C NR	37.7	7.84	9.88	44.6
Pd/CeO_2_/C poly	31.6	6.90	9.91	51.6
Pd/C	X	14.42	10.4	75.2

A comparative analysis of the results obtained from XPS survey spectra, Rietveld quantification, and SEM-EDS was conducted to assess their agreement. All results are presented in Table S1, which summarizes the ratios between Pd/Ce or Pd/CeO_2_. The comparison reveals that the (At% Pd)/(At% Ce) and (At% Pd)/(At% CeO_2_) ratios exhibit similar trends across the different techniques. For Rietveld analysis, the (At% Pd)/(At% CeO_2_) ratio follows a descending order: Pd/CeO_2_/C NS > Pd/CeO_2_/C poly > Pd/CeO_2_/C NR ∼ Pd/CeO_2_/C NC. Similarly, in the SEM-EDS analysis, the (At% Pd)/(At% Ce) ratio decreases in the same order. The XPS data align closely with these trends, further reinforcing the consistency and reliability of the results obtained across all methods.

Figure 5 illustrates XPS core-level spectra of Ce 3d for the catalyst materials. Cerium spin–orbit coupling 3d_5/2_ and 3d_3/2_ are presented as eight characteristic peaks. Three pairs of doublets corresponding to the Ce^4+^ state and one pair for the Ce^3+^ state, indicating the presence of both Ce^4+^ and Ce^3+^ on the ceria surface.^61,96,97^ Table S1 in the Supporting Information detail the positions and areas of the XPS Ce 3d peak components. The relative amount of Ce^4+^ and Ce^3+^ are presented in Table 8 for all catalysts.

**Figure 5:**
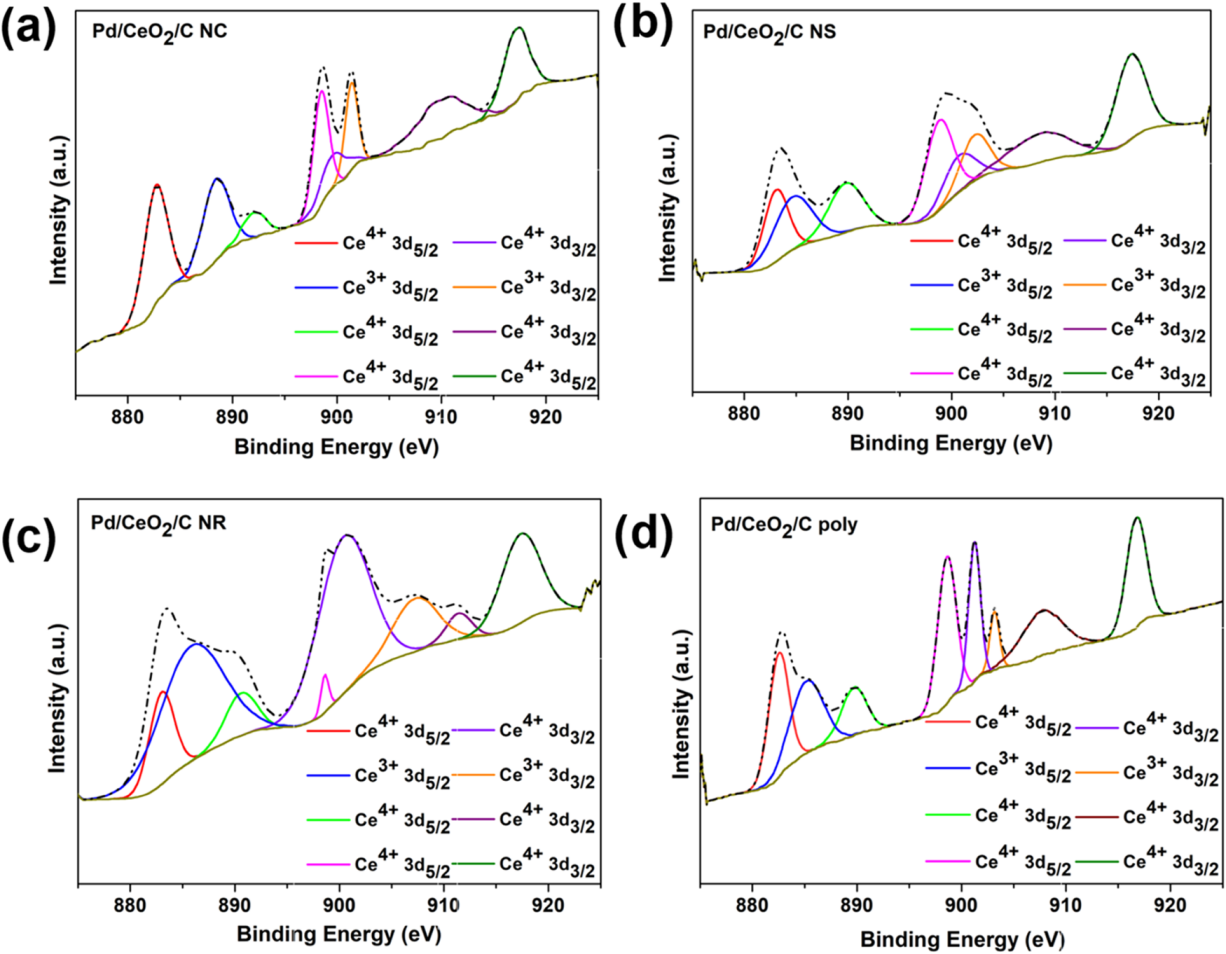
XPS Ce 3d core-level spectra of Pd/CeO_2_/C nanomaterials (a) Pd/CeO_2_/C NC, (b) Pd/CeO_2_/C NS, (c) Pd/CeO_2_/C NR, and (d) Pd/CeO_2_/C poly.

**Table 8:** XPS Results Obtained by Ce 3d, Pd 3d, and O 1s Core-Level Spectra of Catalysts

spectra	at (%)	Pd/CeO_2_/C NC	Pd/CeO_2_/C NS	Pd/CeO_2_/C NR	Pd/CeO_2_/C poly	Pd/C
Ce 3d	Ce^4+^	75.0	77.3	62.4	80.6	X
	Ce^3+^	25.0	22.7	37.6	19.4	X
O 1s	Ce^4+^–O^2–^	52.3	55.31	49.6	54.2	X
	Ce–OH	32.2	29.0	25.5	31.7	X
	Ce^3+^–O^2–^	15.6	15.1	24.9	14.1	X
Pd 3d	Pd^0^	73.3	74.7	75.8	61.7	61.2
	Pd^2+^	26.7	25.3	24.2	38.3	38.8

The O 1s core-level spectra (Figure 6) show three distinct component peaks, corresponding to electron emissions from O 1s associated with Ce^4+^-O^2–^ lattice species, surface-adsorbed species such as hydroxyl groups (Ce–OH), and Ce^3+^-O^2–^ lattice species. The latter is particularly indicative of the presence of oxygen vacancies, which play a crucial role in the material’s catalytic properties.^60,98^ Table S3 in the Supporting Information details the positions and areas of the XPS O 1s peak components. The relative amounts of Ce^4+^–O^2–^, Ce–OH, and Ce^3+^–O^2–^ are presented in Table 8 for all catalysts.

**Figure 6:**
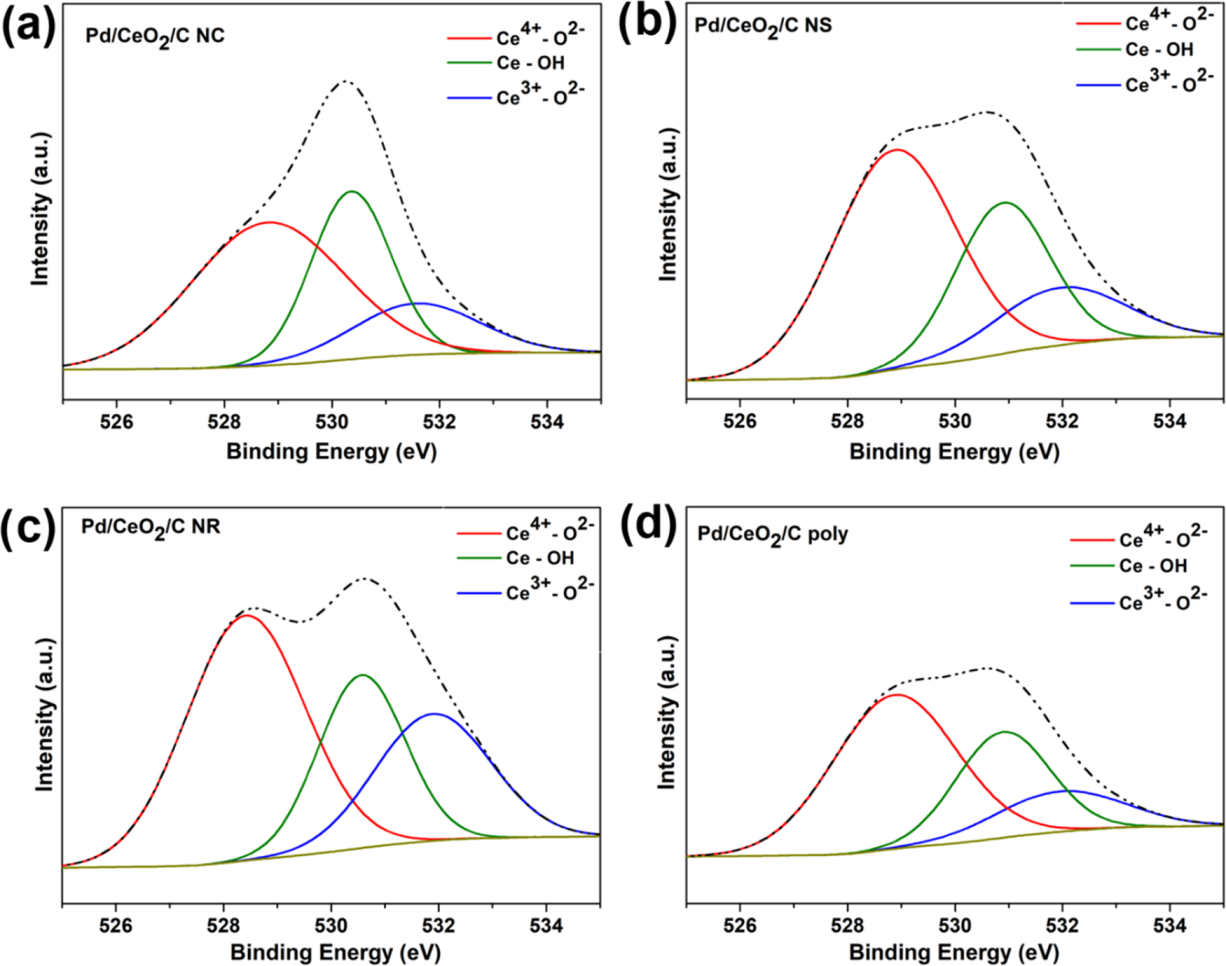
XPS O 1s core-level spectra of Pd/CeO_2_/C nanomaterials (a) Pd/CeO_2_/C NC, (b) Pd/CeO_2_/C NS, (c) Pd/CeO_2_/C NR, and (d) Pd/CeO_2_/C poly.

Figure 7 shows the XPS Pd 3d core-level spectra for all catalysts, showing four component peaks corresponding to the spin–orbit coupling of 3d_5/2_ and 3d_3/2_. One doublet pair represents the Pd^0^ state, while another pair corresponds to the Pd^2+^ state, associated with PdO species, indicating the coexistence of both species.^99–102^ Table S4 in the Supporting Information details the positions and areas of the XPS Pd 3d peak components. The relative amounts of Pd^0^ and Pd^2+^ are presented in Table 8 for all catalysts.

**Figure 7:**
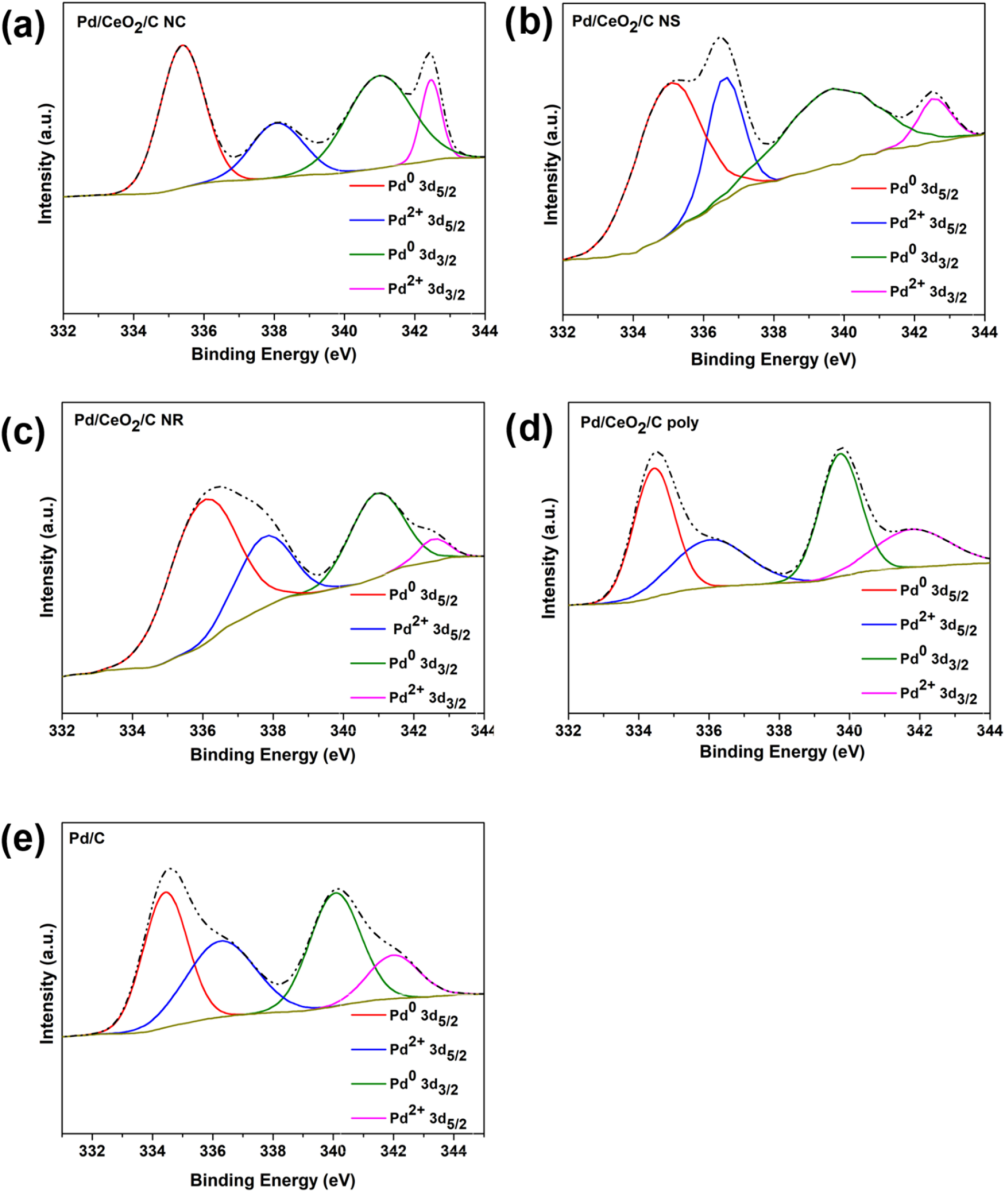
XPS O 1s core-level spectra of Pd/C-CeO_2_ nanomaterials (a) Pd/CeO_2_/C NC, (b) Pd/CeO_2_/C NS, (c) Pd/CeO_2_/C NR, (d) Pd/CeO_2_/C poly, and (e) Pd/C.

The analysis in Table 8 reveals that the relative percentages of Ce^4+^ consistently exceeded those of Ce^3+^ for all ceria NPs, indicating the predominance of Ce^4+^ ions on the surface of Pd/CeO_2_/C nanomaterials.^58,60,61,96,103^ Since XPS analysis showed the presence of Ce^3+^ ions and the absence of a crystalline Ce_2_O_3_ phase was observed in XRD patterns, it is assumed that Ce_2_O_3_ exists in an amorphous state, likely on the surface.^104,105^ The atomic ratio trend for Ce^3+^ in ceria NPs with polyhedral, nanocube, and nanorod morphologies aligns with previously reported values, whereas the observed trend for Pd^2+^ was inverted.^79^ This deviation may be attributed to the lower palladium content deposited on ceria (Pd% ∼ 2%), which enhances the interaction between ceria oxygen and Pd NPs, resulting in a higher proportion of cations in the materials. Furthermore, the increasing order of oxygen vacancy content observed in Table 8 is consistent with trends reported in the literature.^79^

It was observed that Pd/CeO_2_/C NR exhibited the highest Ce^3+^ content, while Pd/CeO_2_/C poly presented the lowest. For Pd/CeO_2_/C NC, the Ce^3+^ content was 1.29 times lower than in Pd/CeO_2_/C NR and 1.10 times higher than in Pd/CeO_2_/C NS. Similarly, the oxygen vacancy content in Pd/CeO_2_/C NC was 1.50 times lower than in Pd/CeO_2_/C NR and 1.09 times higher than in Pd/CeO_2_/C NS. The Ce^3+^ and oxygen vacancies relative amount followed the order Pd/CeO_2_/C NR, Pd/CeO_2_/C NC, Pd/CeO_2_/C NS, and Pd/CeO_2_/C poly. Additionally, Pd/CeO_2_/C NC showed the highest concentration of surface-adsorbed OH species, whereas Pd/CeO_2_/C NR had the lowest. The OH content in Pd/CeO_2_/C poly was 0.93 times lower than in Pd/CeO_2_/C NC but 1.06 times higher than in Pd/CeO_2_/C NS. The relative amount of OH adsorbed species in ceria NPs was following Pd/CeO_2_/C NC, Pd/CeO_2_/C poly, Pd/CeO_2_/C NS, and Pd/CeO_2_/C NR.

Moreover, Pd/CeO_2_/C NR showed the highest amount of Pd^0^, while Pd/C presented the lowest amount. The Pd^0^ relative amount followed the order Pd/CeO_2_/C NR, Pd/CeO_2_/C NS, Pd/CeO_2_/C NC, Pd/CeO_2_/C poly, and Pd/C. Incorporating ceria NPs into palladium-based materials increased the Pd^0^ percentage in Pd/CeO_2_/C samples compared to Pd/C. Hence, it is inferred that the presence of ceria NPs in the catalysts results in an increase in the amount of Pd^0^. Moreover, the morphology of ceria significantly influences the amount of Pd^0^ in the catalysts. Materials containing ceria with controlled morphology exhibited a higher concentration of Pd^0^ compared to those with polyhedral ceria.

A comparison of the XPS data (Table 8) with XRD results (Table 3) for ceria NPs in Pd/C-CeO_2_ reveals a similar tendency in the relative percentage of Ce^3+^, the unit cell lattice parameter, and the oxygen vacancy content. Furthermore, an increase in Pd^0^ concentration was observed with the rising amount of dislocation density. This correlation is anticipated, as a greater density of surface defects offers additional anchoring sites for Pd^2+^ during the synthesis process, thereby enhancing its reduction to metallic palladium.^93^

Considering that all ceria-containing nanocomposites—except for Pd/CeO_2_/C NR—exhibited similar surface concentrations of oxygen vacancies, the determining factor for the intensity of the metal–support interaction (MSI)^106–110^ in these materials would be the microstrain of the ceria crystal lattice and the reactivity of the preferentially exposed surface. In Pd/CeO_2_/C poly, ceria nanoparticles predominantly expose the (111) plane,^93^ which is thermodynamically stable, nonpolar, and less prone to the formation or accommodation of oxygen vacancies.^25,111^ XRD analysis reveals a slight tensile microstrain, suggesting weaker overlap between Ce_5*d*/4f_ and O_2p_ orbitals, leading to weakened Ce–O bonds^76–78^ and reduced vacancy mobility. Therefore, it is inferred that the MSI in this system is the weakest among the analyzed materials.

For the Pd/CeO_2_/C NC nanocomposite, which predominantly exposes the (100) plane^28–31^ —polar, highly reactive, and favorable for the formation of oxygen vacancies but with low stability^25,111^ —a pronounced tensile microstrain is observed, indicating increased vacancy mobility. Given the high surface reactivity, the MSI intensity in this material is presumably the strongest.

In the case of Pd/CeO_2_/C NS, the nanoparticles preferentially expose the (110) plane,^34,35^ which exhibits intermediate reactivity, moderate ease of oxygen vacancy formation, and high capacity for vacancy accommodation.^25,111^ This morphology is accompanied by the highest tensile microstrain observed, suggesting highly mobile and stable vacancies. Therefore, the MSI in this material is considered intermediate.

Finally, in Pd/CeO_2_/C NR, ceria nanoparticles predominantly expose the (100) and (110) planes,^28–31^ conferring a combination of moderate to high surface reactivity and polar character. This material presents the highest concentration of oxygen vacancies—approximately twice that of the other samples. However, the presence of compressive microstrain indicates a rigid lattice due to enhanced orbital overlap between Ce_5*d*/4f_ and O_2p_, strengthening the Ce–O bonds,^76–78^ thereby limiting vacancy mobility and consequently restricting MSI intensity despite the high defect density. As a result, Pd/CeO_2_/C NR likely exhibits the second weakest MSI effect, stronger only than that of Pd/CeO_2_/C poly.

### 3.2 Electrochemical Results

Figure 8 shows cyclic voltammograms (CV) of the electrocatalysts in 1 mol L^–1^ NaOH at a scan rate of 50 mV s^–1^ from −0.85 to 0.10 V.^22^ The direction of the forward scan is indicated by the red arrow, while the direction of the reverse scan is marked by the blue arrow. During the direct scan, an oxidation peak corresponding to hydrogen desorption (eq 6) is observed on the Pd(111) and Pd(100) planes at approximately −0.73 and −0.51 V, respectively.^112^ Additionally, hydroxyl group adsorption occurs around −0.31 V (eq 7),^22,113^ and a Pd–O layer forms at approximately −0.01 V (eq 8).^113^ In the reverse scan, reduction peaks associated with Pd–O layer reduction appear at about −0.20 V (eq 9),^113^ and a region of hydrogen adsorption is observed from −0.62 to −0.73 V (eq 10),^112^ originating from the water activation reaction (eq 11).^114^

6 $$Pd-H_{ads}\to Pd+H_{\left(aq\right)}^{+}+e^{-}$$

7 $$\mathrm{Pd}+{\mathrm{OH}}_{\left(\mathrm{aq}\right)}^{-}\to \mathrm{Pd}-{\mathrm{OH}}_{\mathrm{ads}}+e^{-}$$

8 $$\mathrm{Pd}-{\mathrm{OH}}_{\mathrm{ads}}+{\mathrm{OH}}_{\left(\mathrm{aq}\right)}^{-}\to \mathrm{Pd}-O+e^{-}$$

9 $$\mathrm{Pd}-O+H_{2}O+2e^{-}\to \mathrm{Pd}+2{\mathrm{OH}}_{\left(\mathrm{aq}\right)}^{-}$$

10 $$\mathrm{Pd}+H_{\left(\mathrm{aq}\right)}^{+}+e^{-}\to \mathrm{Pd}-H_{\mathrm{ads}}$$

11 $$H_{2}O⇌H_{\left(\mathrm{aq}\right)}^{+}+e^{-}+{\mathrm{OH}}_{\mathrm{ads}}$$

**Figure 8:**
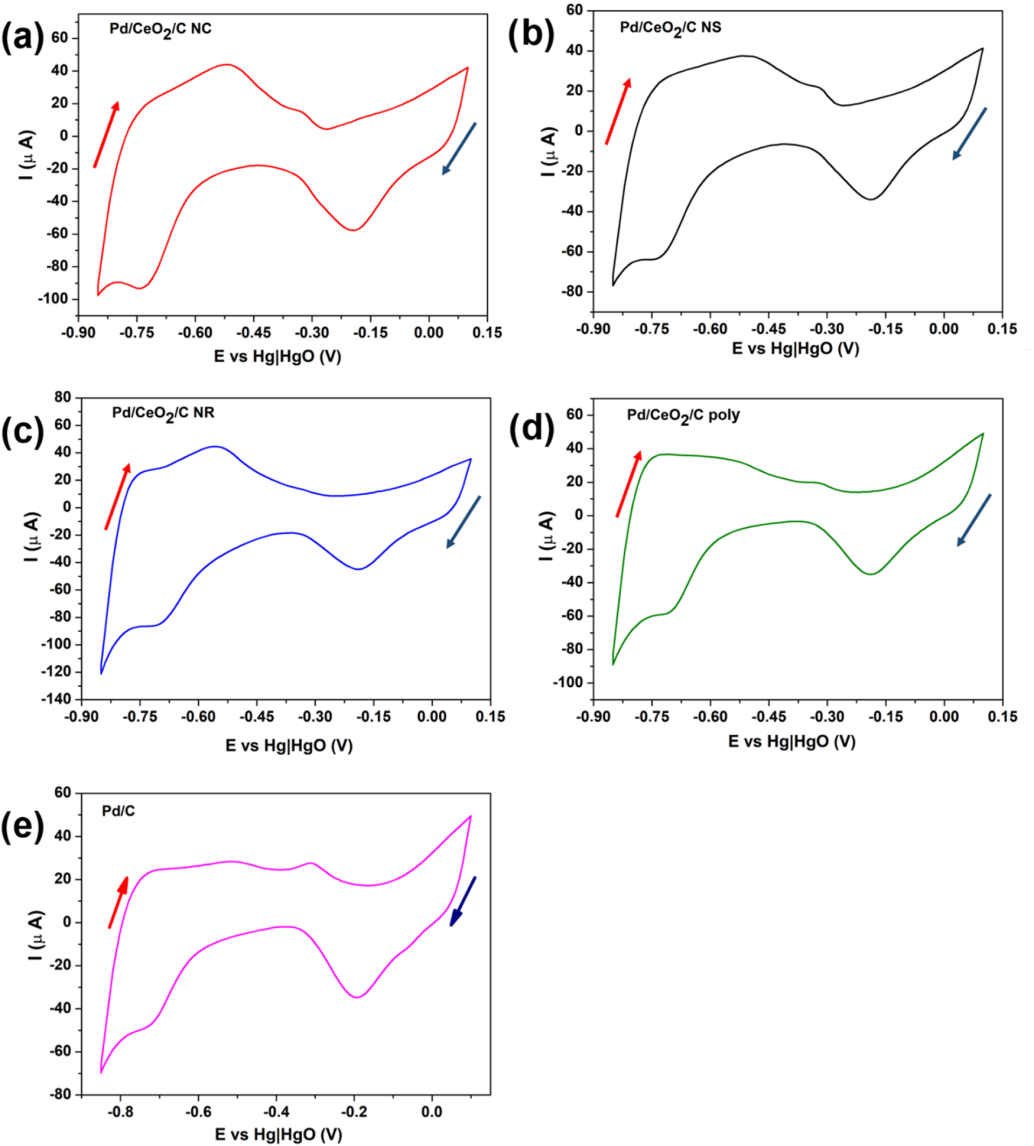
Cyclic voltammograms in 1 mol L^–1^ NaOH at 50 mV s^–1^ and at 300 K of (a) Pd/CeO_2_/C NC, (b) Pd/CeO_2_/C NS, (c) Pd/CeO_2_/C NR, (d) Pd/CeO_2_/C poly, and (e) Pd/C.

When comparing the voltammograms presented in Figure 8, it can be observed that electrocatalysts containing ceria with controlled morphology exhibit a more defined Pd–H desorption peak compared to Pd/C and Pd/CeO_2_/C poly. This observation suggests a more facilitated hydrogen desorption for Pd/CeO_2_/C NR, and a less facilitated hydrogen desorption for Pd/C. It is proposed that the hydrogen desorption from Palladium surface follows the order Pd/CeO_2_/C NR, Pd/CeO_2_/C NC, Pd/CeO_2_/C NS, Pd/CeO_2_/C poly, and Pd/C.

Formate electrooxidation proceeds via three mechanisms: a direct dissociative path, a direct associative path, and an indirect path.^115^ Scheme 1 shows the mechanism of formate electrooxidation. In the direct dissociative path, adsorbed formate species are directly oxidized to CO_2_ via dehydrogenation reactions (eqs 12–14).^115^ In the direct associative path, adsorbed formate reacts with OH species to form COO^–^ adsorbed species (eq 15), which then produce CO_2_ (eq 13).^115^ In the indirect path, adsorbed formate species lead to CO_(ads)_ through dehydration (eq 16),^24,114^ which subsequently oxidizes to CO_2_ with the assistance of OH species (eq 17).^24,115^

**Scheme 1:**
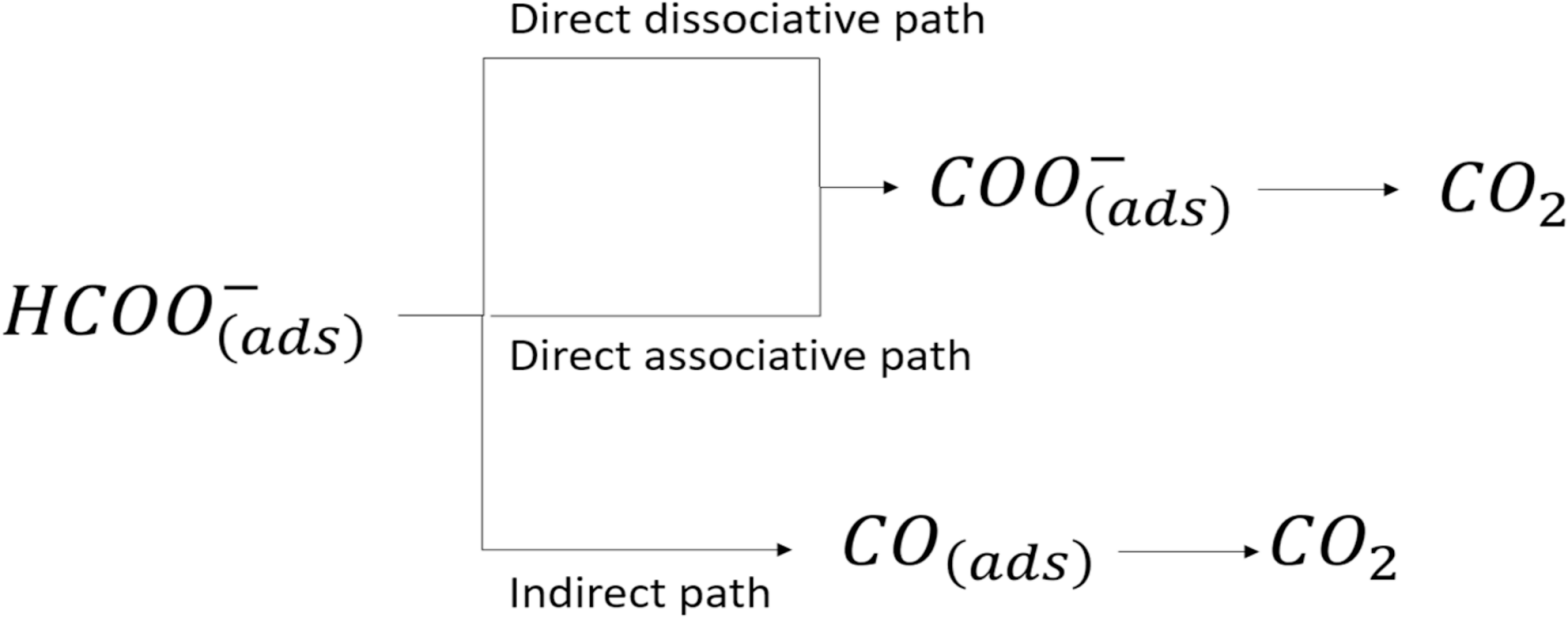
Schematic Representation of the Formate Electrooxidation Process

**Direct dissociative path**

12 $${\mathrm{HCOO}}_{\left(\mathrm{ads}\right)}^{-}\to {\mathrm{COO}}_{\left(\mathrm{ads}\right)}^{-}+H_{\left(\mathrm{ads}\right)}$$

13 $${\mathrm{COO}}_{\left(\mathrm{ads}\right)}^{-}\to {\mathrm{CO}}_{2\left(\mathrm{ads}\right)}+e^{-}$$

14 $$H_{\left(\mathrm{ads}\right)}+{\mathrm{OH}}_{\left(\mathrm{aq}\right)}^{-}\to H_{2}O_{\left(\mathrm{ads}\right)}+e^{-}$$

**Direct associative path**

15 $${\mathrm{HCOO}}_{\left(\mathrm{ads}\right)}^{-}+OH_{\left(\mathrm{ads}\right)}\to {\mathrm{COO}}_{\left(\mathrm{ads}\right)}^{-}+H_{2}O_{\left(\mathrm{ads}\right)}$$

**Indirect path**

16 $${\mathrm{HCOO}}_{\left(\mathrm{ads}\right)}^{-}+H_{\left(\mathrm{aq}\right)}^{+}\to {\mathrm{CO}}_{\left(\mathrm{ads}\right)}+H_{2}O_{\left(\mathrm{ads}\right)}$$

17 $${\mathrm{CO}}_{\left(\mathrm{ads}\right)}+{2\mathrm{OH}}_{\left(\mathrm{aq}\right)}^{-}\to {\mathrm{CO}}_{2\left(\mathrm{ads}\right)}+H_{2}O_{\left(\mathrm{ads}\right)}+2e^{-}$$

Figure 9 shows cyclic voltammograms obtained in 1 mol L^–1^ NaOH and 1 mol L^–1^ HCOONa at a scan rate of 20 mV s^–1^. The direction of the forward scan is indicated by the red arrow, while the reverse scan direction is marked by the blue arrow. The forward scan exhibits two oxidation peaks, while the backward scan shows one oxidation peak.

**Figure 9:**
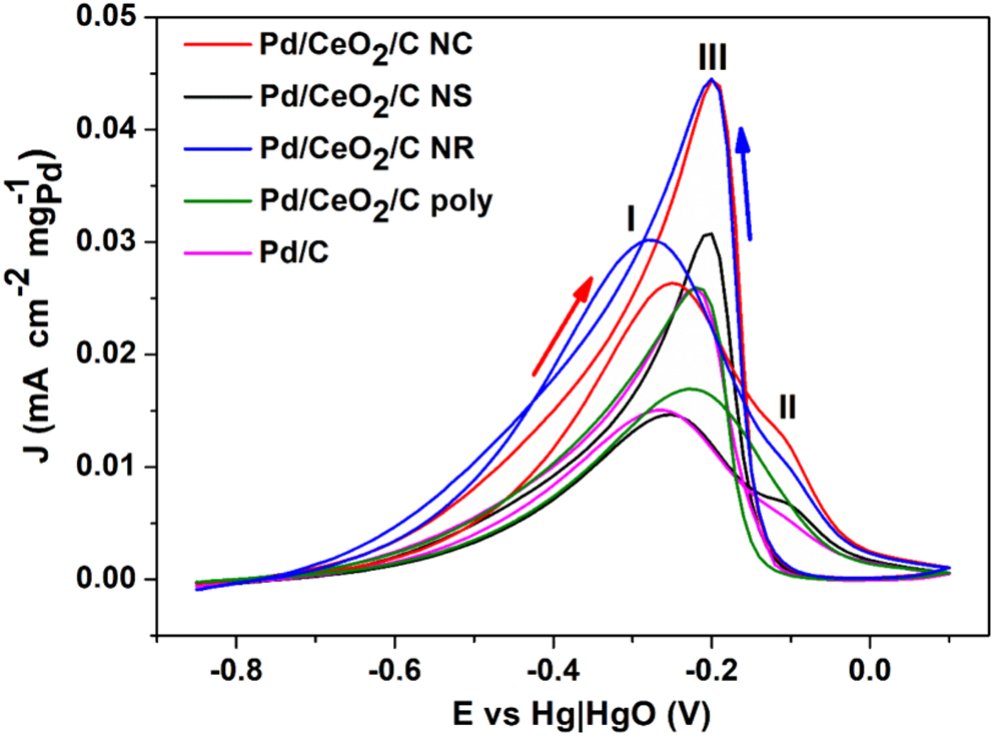
Cyclic voltammograms in 1 mol L^–1^ NaOH + 1 mol L^–1^ HCOONa at 20 mV s^–1^ and room temperature.

In the forward scan, the lower onset potential indicates an increase in the dissociative adsorption of COO^–^ molecules, leading to a higher peak current density at lower overpotentials.^116^ In the forward scan, the formate reaction begins at the hydrogen desorption potential of the Pd(100) plane, suggesting that formate preferentially adsorbs on this plane, where the dissociative pathway is favored. The lower onset potential for the reaction indicates a preference for the dissociative adsorption of COO^–^ molecules, leading to a higher peak current density at lower overpotentials.^116,117^ The onset potentials for the FER of the materials are approximately −0.54, −0.47, −0.42, −0.44, and −0.42 V for Pd/CeO_2_/C NR, Pd/CeO_2_/C NC, Pd/CeO_2_/C NS, Pd/CeO_2_/C poly, and Pd/C, respectively. These results suggest that the direct dissociative pathway is favored in the following order: Pd/CeO_2_/C NR shows the highest favorability for the direct dissociative pathway, followed by Pd/CeO_2_/C NC, Pd/CeO_2_/C poly, Pd/CeO_2_/C NS, and Pd/C, which shows the lowest favorability.

The contribution to the formation of peak I in the forward scan likely arises from both the direct associative and dissociative pathways.^24,115,118–120^ The current density continues to increase as hydroxyl adsorption occurs on the Pd surface. At this stage, peak I receive the contribution from the direct associative pathway. The current density of peak I reaches its maximum upon the completion of the Pd–OH layer. The Pd–OH saturation suppresses the direct dissociative mechanism while favoring the direct associative mechanism,^119^ resulting in a subsequent decrease in current density. These observations indicate that the direct dissociative mechanism is the rate-determining step of the FER on the Pd surface. The formation of the Pd–O layer progressively suppresses the reactions contributing to peak I. Direct mechanisms are fully inhibited once the Pd–O layer is completely formed.^119^ The maximum current density for peak I during FER increases by factors of 1.79, 1.57, and 1.13 for Pd/CeO_2_/C NR, Pd/CeO_2_/C NC, and Pd/CeO_2_/C poly, respectively, compared to Pd/C. In contrast, Pd/CeO_2_/C NS exhibits a decrease in peak current density by a factor of 0.86 relative to Pd/C.

A well-defined peak for CO_(ads)_ oxidation is not observed in Pd/C, whereas this peak becomes evident and is denoted as peak II for ceria-containing materials. The formation of peak II relies on the presence of the Pd–OH layer and is inhibited by the formation of the Pd–O layer.^115,121^ All ceria-containing catalysts exhibited a more defined peak II, indicating that the addition of ceria NPs promotes the oxidation of CO adsorbed on the palladium surface. According to eq 16, carbon monoxide formation is promoted by the presence of Pd–H.^24,114^ Therefore, we propose that the faster the Pd–H desorption, the lower the potential formation of CO, and the higher the catalytic activity of the electrocatalyst. This is particularly relevant since carbon monoxide is considered a catalytic poison due to its strong adsorption on active sites, leading to their deactivation.^17,122,123^

The electrocatalyst with the most well-defined peak II was Pd/CeO_2_/C NS, followed by Pd/CeO_2_/C NC, Pd/CeO_2_/C NR, Pd/CeO_2_/C poly, and Pd/C. This observation suggests a decreasing of CO oxidation across the materials. However, this trend does not directly imply diminished catalyst poisoning, as a high CO oxidation rate to CO_2_ may be accompanied by an equally high CO formation rate, leading to substantial CO adsorption on the palladium surface and subsequent poisoning.

In cyclic voltammetry, catalyst poisoning can be assessed using the ratio of the maximum current densities ***J***_***max***_ of peaks III and I, expressed as ***J***_***maxIII***_ **/** ***J***_***maxI***_.^24,56^ In the backward scan, peak III emerges as the Pd–O layer begins to reduce, suggesting that the primary contribution to peak III arises from direct dissociative reactions. These reactions depend on the palladium surface being unblocked.^119^ Thus, a higher ***J***_***maxIII***_ **/** ***J***_***maxI***_ ratio may indicate a greater amount of CO produced during the formation of peak I, a lower capacity for CO oxidation during peak II, and, consequently, an increased extent of catalytic poisoning.^24,56^ The calculated values for ***J***_***maxIII***_ **/** ***J***_***maxI***_ were 1.66, 2.10, 1.46, 1.73, and 1.52 for Pd/CeO_2_/C NC, Pd/CeO_2_/C NS, Pd/CeO_2_/C NR, Pd/CeO_2_/C poly, and Pd/C, respectively. These results align with the previously established conclusions. Accordingly, the decrease in catalytic activity followed the order Pd/CeO_2_/C NR, Pd/CeO_2_/C NC, Pd/CeO_2_/C poly, Pd/CeO_2_/C NS, and Pd/C.

The electrocatalytic activity of Pd/CeO_2_/C nanocomposites in formate electrooxidation reaction under alkaline conditions is proposed to arise from the synergy between two structural factors: the crystallographic nature of CeO_2_ (exposed planes, reactivity, and vacancy mobility),^76–78^ and the intensity of the metal–support interaction (MSI).^106–110^ The mechanism governing the charge transfer from ceria to palladium can be understood as a combination of lattice tensile strain in CeO_2_ (which weakens Ce–O bonds and facilitates vacancy mobility),^76–78^ the reactivity of the exposed crystallographic planes (which influences the ease of forming reactive oxygen vacancies),^25,111^ and the intensity of the MSI effect (which could enables efficient electronic transfer to Pd).^106–110^

These parameters act synergistically to modulate the electronic density at the Pd surface, directly influencing the adsorption and desorption steps of reaction intermediates. Thus, each system exhibits distinct behavior in light of the Sabatier principle, which postulates that optimal catalytic activity is achieved when the interaction between the catalyst and adsorbed species is moderate—strong enough to ensure effective chemisorption, but weak enough to allow rapid desorption of products.^124,125^ Excessively strong interactions between Pd and intermediates can lead to site blocking due to irreversible adsorption, resulting in catalyst poisoning. Conversely, interactions that are too weak compromise intermediate stability at the surface, hindering reactant activation and limiting overall process efficiency.^124,125^

Under anodic polarization, the electric field can induce the migration of oxygen vacancies in CeO_2_.^126^ When combined with an appropriately intense MSI effect, this could promote effective electron donation from ceria to Pd,^106–110^ leading to modifications in the surface chemical environment and reconfiguration of Pd’s valence electronic structure,^127^ thereby increasing the dipole charge on its surface.^87^ In this context, it is proposed that CeO_2_ nanoparticles with different morphologies exhibit distinct abilities to form new oxygen vacancies and, due to varying MSI effects, different capacities to donate electronic density to Pd, thereby modifying the Pd surface energy and the adsorption/desorption energies during electric field application.

Guo et al.^87^ investigated the synergy between compressive strain and oxygen vacancies on the catalytic activity of PdCu nanoparticles supported on CeO_2_ nanorods, and observed that an intense dipole charge on the palladium surface could lead to a stronger electrostatic interaction between palladium and polar molecules, thereby decreasing the adsorption and desorption energies.^87^ Chen et al.^128^ studied the low-temperature oxidation of formaldehyde (HCHO) using Pd/CeO_2_ catalysts with different morphologies—nanocube, nanorod, and commercial—demonstrating that Pd/CeO_2_ nanocubes exhibited the highest catalytic activity due to the strong Pd/CeO_2_ interfacial interaction, which ensured high Pd dispersion and stability, resulting in complete HCHO conversion to CO_2_ and H_2_O at room temperature.^128^

In Pd/CeO_2_/C NC, the predominant exposure of the (100) plane imparts CeO_2_ with high reactivity, polarity, and propensity to generate unstable (highly reactive) oxygen vacancies.^25,111^ When combined with significant tensile microstrain in the CeO_2_ lattice, this configuration favors the formation of mobile oxygen vacancies.^76–78^ Together with a strong MSI, this environment leads to robust charge transfer from ceria to Pd,^106–110^ promoting a negatively charged Pd surface (strong dipole), which results in high adsorption/desorption energy—yet not excessively high—reflected in the second-best catalytic performance among the samples.

In Pd/CeO_2_/C NS, although the ceria lattice tensile strain is similar to that of Pd/CeO_2_/C NC, the exposure of the (110) plane in nanosheet CeO_2_ leads to an intermediate surface reactivity and more stable vacancies.^25,111^ Combined with a moderate MSI effect, the system might exhibits less efficient charge transfer from CeO_2_ to Pd,^106–110^ which may result in a lower negative charge on the Pd surface. A weakened surface dipolein Pd could diminish formate affinity, hindering molecule adsorption and decreasing the catalytic activity. Thus, this material deviates from the ideal Sabatier conditions for formate adsorption and desorption on Pd, resulting in the lowest catalytic activity among the samples.

For Pd/CeO_2_/C NR, the combination of (100) and (110) planes provides a moderately reactive surface.^25,111^ The CeO_2_ lattice shows compressive microstrain, which partially decreases vacancy mobility.^76–78^ However, the oxygen vacancy density in CeO_2_ NR NPs is approximately twice that of the other samples. This structural condition, coupled with an intermediate MSI intensity, might yield a substantial electronic reservoir, enabling moderate charge transfer from ceria to Pd under electric field application. This results in a Pd surface with intermediate dipole intensity, favoring formate adsorption and efficient desorption of reaction intermediates. Consequently, Pd/C-CeO_2_ NR exhibits the highest electrocatalytic activity.

In Pd/CeO_2_/C poly, the exposure of the (111) plane—apolar and stable surface—limits CeO_2_ surface reactivity and hampers the formation of reactive vacancies.^25,111^ Additionally, both lattice microstrain and MSI effect are the weakest among the composites, which may restrict electronic transfer from ceria to Pd. The insufficient electron density supplied by CeO_2_ poly might hinder the development of a favorable surface dipole for formate adsorption, resulting in poor catalytic activity. Therefore, the XRD, XPS, and magnetization results, which provided detailed insights into ceria structure and MSI intensity, are consistent with the Sabatier principle and support the findings obtained from cyclic voltammetry analysis.

Figure 10 shows the chronoamperometric measurements performed at −0.55 V in NaOH 1 mol L^–1^ + HCOONa 1 mol L^–1^ for 30 min.^24^ A significant decrease in current density from the formate electrooxidation reaction was observed over all catalysts in the first few minutes, followed by stabilization over time. The current density for formate electrooxidation after 30 min was 3.68 times higher for Pd/CeO_2_/C NR compared to Pd/C. For Pd/CeO_2_/C NC, and Pd/CeO_2_/C NS, the current density was 1.98 and 1.49 times higher than that of Pd/C, respectively, while Pd/CeO_2_/C poly was 0.78 times lower than Pd/C. These results are consistent with those observed in cyclic voltammetry, as well as with the physicochemical characterizations, and indicate superior durability of Pd/CeO_2_/C NR.^129^

**Figure 10:**
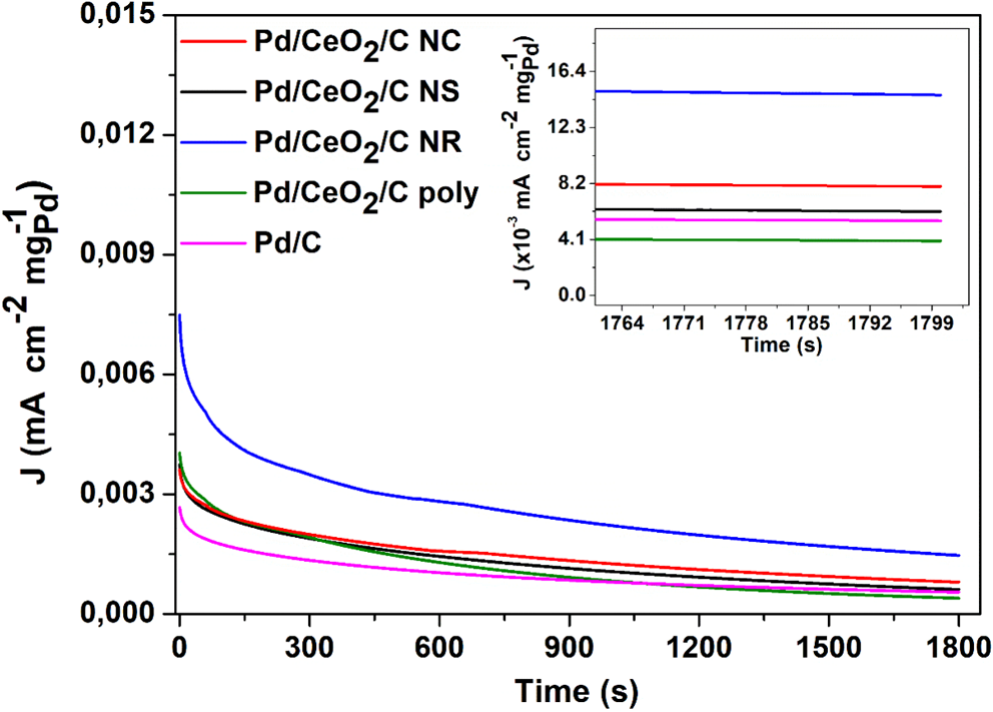
Chronoamperometric measurements at −0.55 V vs Hg|HgO for of Pd/C and Pd/CeO_2_/C electrocatalysts in 1 mol L^–1^ NaOH + 1 mol L^–1^ HCOONa at room temperature. The inset figure presents the last 50 s of the experiment.

The high catalytic stability observed for the Pd/CeO_2_/C NR material is suggested to result from the synergistic combination of a high density of moderately mobile oxygen vacancies (due to compressive microstrain) on the CeO_2_ surface and an intermediate-strength MSI, which may facilitate charge transfer from ceria to palladium and the formation of a sufficiently negative surface dipole on Pd. This configuration optimizes formate adsorption and product desorption even under prolonged polarization, leading to enhanced temporal stability of the current. In contrast, the low catalytic stability of Pd/CeO_2_/C poly might be attributed to the ceria structure with low surface reactivity, limited ability to supply electronic density to palladium, and a weaker MSI. These factors result in a reduced electron transfer from ceria to Pd during formate electrooxidation and, consequently, a more pronounced decline in catalytic stability over time.

In general, the presence of oxygen vacancies in CeO_2_ materials is known to enhance the catalytic activity of noble metal electrocatalysts.^130,131^ The presence of oxygen vacancies is typically correlated with increased catalytic activity and poisoning tolerance. However, as indicated by the results of cyclic voltammetry and chronoamperometry, and supported by the findings from XPS and XRD, the presence of oxygen vacancies in Pd/CeO_2_/C catalysts may not be the sole determining factor influencing the enhanced catalytic activity compared to Pd/C. The interaction between Palladium and ceria, oxygen vacancy concentration, and crystal defects in nanoparticles likely plays a crucial role in facilitating the adsorption and desorption of adsorbates on the palladium surface, thereby favoring direct pathways in the formate electrooxidation reaction.

## 4 Conclusions

In this study, the electrocatalytic activity of Pd/CeO_2_/C NR catalysts with CeO_2_ nanoparticles of different morphologies (cubes, hexagonal sheets, and nanorods) was evaluated for formate electrooxidation reactions in alkaline media. Microscopy images revealed that the ceria nanoparticles exhibited well-defined morphology, with palladium nanoparticles having average sizes of 4 to 10 nm. Rietveld refinement of diffraction patterns indicated that Pd/CeO_2_/C NR exhibited larger lattice parameters, along with compressive microstrain and higher defect values compared to other catalysts. XPS analysis further revealed a higher amount of oxygen vacancies and a greater proportion of metallic palladium nanoparticles in Pd/CeO_2_/C NR. Among the materials studied, Pd/CeO_2_/C NR demonstrated the highest catalytic activity for FER. The maximum peak current density observed in CV analysis from formate electrooxidation on Pd/CeO_2_/C NR and Pd/CeO_2_/C NC was 1.79 and 1.57 times greater, respectively, than that observed on Pd/C. In contrast, Pd/CeO_2_/C NS exhibits a peak current density that is 0.86 times lower than that of Pd/C. In CA measurements, the current density from FER on Pd/CeO_2_/C NR is 2.65 times higher than that on Pd/C, while for Pd/CeO_2_/C NC and Pd/CeO_2_/C NS, the current density is 1.45 and 1.13 times higher, respectively, compared to Pd/C. This enhanced catalytic activity and stability could be attributed to the synergistic interaction between palladium and ceria, involving compressive microstrain and a high concentration of oxygen vacancies in the CeO_2_ NR nanoparticles, an intermediate MSI effect, and a significant surface dipole charge on Pd. These factors collectively facilitate the adsorption of polar adsorbates, such as water and formate, on the palladium nanoparticles, thereby promoting a higher catalytic activity. These factors contributed to Pd/CeO_2_/C NR’s superior catalytic activity, as evidenced by its higher peak current density in cyclic voltammetry and increased initial and final current densities in chronoamperometry. Therefore, Pd/CeO_2_/C NR emerges as a promising catalyst for direct formate fuel cell applications.

## Supplementary Material



## Notes

Fitting analysis for the Scherrer and SSP methods applied to ceria nanoparticles in electrocatalysts (Figure S1); fitting analysis for the Scherrer and SSP methods applied to palladium nanoparticles in electrocatalysts (Figure S2); comparison of semiquantitative analysis by XRD, XPS, and EDS (Table S1); area and position of individual peak components of Ce 3d for catalyst nanomaterials (Table S2); area and position of individual peak components of O 1s peaks for catalyst nanomaterials (Table S3); and area and position of individual peak components of Pd 3d peaks for catalyst nanomaterials (Table S4) (PDF)
