# Supplementary material for: Influence of CeO2 Nanoparticle Morphology on the Electrocatalytic Activity of Palladium toward the Formate Electrooxidation Reaction
Source: ACS Omega. 2025 Aug 15;10(33):37830–48. doi: 10.1021/acsomega.5c04822 (PMC12392020; doi:10.1021/acsomega.5c04822)
Supplement: Supplementary file 1 [file ao5c04822_si_001.pdf]

SUPPORTING INFORMATIONS

The influence of CeO_2 nanoparticle morphology on the electrocatalytic activity of palladium toward formate electrooxidation reaction

Aila O. Santos[a], Giulia K. Silva[a], Hozana S. C. Oliveira[a], Noemi R. C. Huaman[b], André V.H. Soares[c], Odivaldo C. Alves[a], Júlio César M. Silva[a] *

[a]*Departamento de Físico-Química, Instituto de Química, Universidade Federal Fluminense, Campus Valonguinho, 24020-141, Niterói, RJ, Brasil*
[b]*Centro Brasileiro de Pesquisas Físicas, Urca, 22290-180 Rio de Janeiro, RJ, Brasil*
[c]*Departamento de Engenharia Química e de Petróleo, Universidade Federal Fluminense, Campus da Praia Vermelha, 24210-240, Niterói, RJ, Brasil*

- XRD measurements

Figures S1 and S2 illustrate the linear fittings for the Scherrer and SSP models applied to ceria and palladium NPs, respectively. The SSP model demonstrated superior accuracy, with R^2 values closer to 1 compared to the Scherrer model, highlighting its greater reliability and suitability for analyzing the structural properties of the NPs.

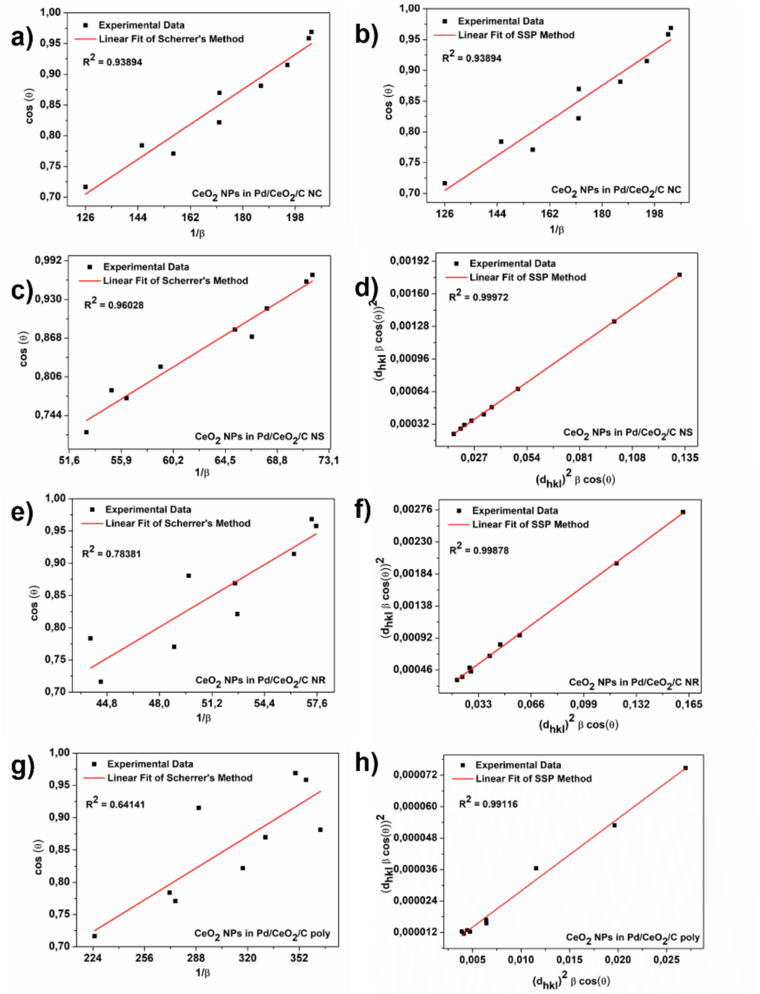

Figure S1. Fitting analysis for the Scherrer and SSP methods applied to ceria nanoparticles in electrocatalysts **(a-b)** Pd/CeO_2/C NC, **(c-d)** Pd/CeO_2/C NS, **(e-f)** Pd/CeO_2/C NR, **(g-h)** Pd/CeO_2/C poly.

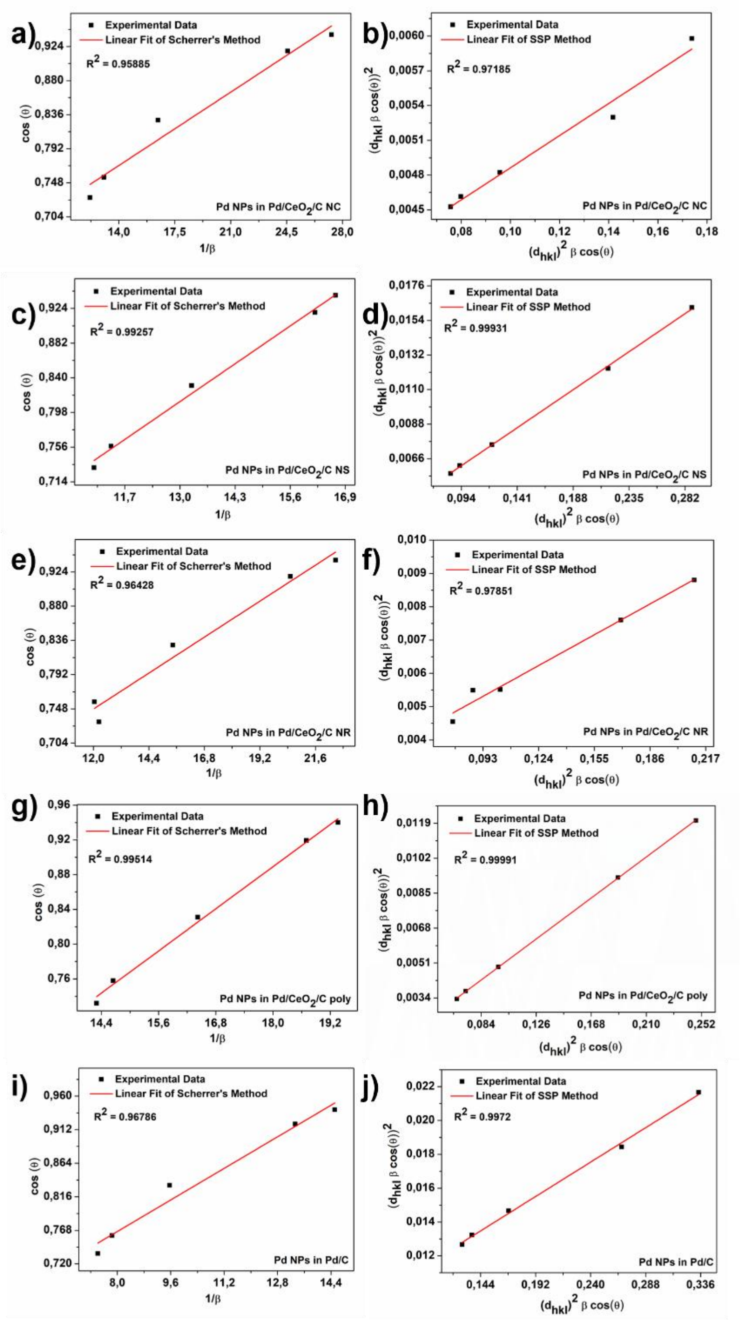

Figure S2. Fitting analysis for the Scherrer and SSP methods applied to Palladium nanoparticles in electrocatalysts **(a-b)** Pd/CeO_2/C NC, **(c-d)** Pd/CeO_2/C NS, **(e-f)** Pd/CeO_2/C NR, **(g-h)** Pd/CeO_2/C poly, **(i-j)** Pd/C.

- XPS core level Ce 3d

A comparison between the results obtained from XPS survey spectra, Rietveld quantification, and SEM-EDS analysis was conducted to evaluate their correspondence. Table 8 summarizes the (At% Pd)/(At% Ce) ratios for each analysis.

Table S1. Comparison of semi-quantitative analysis by XRD, XPS, and EDS.

	**DRX** ($Pd\%/\mathbf{CeO_2}\%$)	**EDS** ($Pd\%/Ce\%$)	**XPS** ($Pd\%/Ce\%$)
**Pd/CeO₂/C NC**	0.21	0.27	0.27
**Pd/CeO₂/C NS**	0.34	0.36	0.36
**Pd/CeO₂/C NR**	0.23	0.26	0.26
**Pd/CeO₂/C poly**	0.26	0.32	0.31

Tables S2-S4 provides detailed information derived from the XPS spectra shown in Figures 5-7. It includes the binding energies, relative intensities, and assignments of the peaks for Ce 3d, O 1s, and Pd 3d. The tables also indicate the chemical states and the proportions of different oxidation states present in the catalyst, offering a comprehensive understanding of the surface composition and electronic structure of the material.

Table S2. Area and position of individual peak components of Ce 3d for catalyst nanomaterials.

	**Pd/CeO₂/C NC**		**Pd/CeO₂/C NS**		**Pd/CeO₂/C NR**		**Pd/CeO₂/C poly**	
**Peak**	**Position (eV)**	**Area (%)**	**Position (eV)**	**Area (%)**	**Position (eV)**	**Area (%)**	**Position (eV)**	**Area (%)**
Ce^{4+} $3d_{5/2}$	882.64	21.19	883.04	11.75	883.00	8.69	882.53	16.45
Ce^{3+} $3d_{5/2}$	888.36	16.47	884.63	13.85	885.82	28.41	885.16	15.76
Ce^{4+} $3d_{5/2}$	892.07	4.37	889.77	11.44	890.60	5.04	889.70	7.99
Ce^{4+} $3d_{5/2}$	898.46	13.61	898.79	16.89	898.61	1.16	898.60	18.03
Ce^{4+} $3d_{3/2}$	899.56	6.80	900.72	7.89	900.37	31.48	901.24	10.92
Ce^{3+} $3d_{3/2}$	901.38	8.53	902.23	8.88	907.24	9.19	903.16	3.68
Ce^{4+} $3d_{3/2}$	909.91	14.64	908.51	11.69	911.41	2.53	907.69	12.30
Ce^{4+} $3d_{3/2}$	917.10	14.39	917.27	17.61	917.33	13.49	916.72	14.84

Table S3. Area and position of individual peak components of O 1s peaks for catalyst nanomaterials.

	$Pd/CeO_2/C$ NC		$Pd/CeO_2/C$ NS		$Pd/CeO_2/C$ NR		$Pd/CeO_2/C$ poly	
**Peak**	**Position (eV)**	**Area (%)**	**Position (eV)**	**Area (%)**	**Position (eV)**	**Area (%)**	**Position (eV)**	**Area (%)**
**Ce^{4+} - O^{2-}**	528.81	52.25	528.88	55.31	528.41	49,58	528.19	54.20
**Ce - OH**	530.36	32.16	530.89	29.05	530.56	25.49	530.56	31.74
**Ce^{3+} - O^{2-}**	531.56	15.59	531.95	15.13	531.88	24.93	531.66	14.06

Table S4. Area and position of individual peak components of Pd 3d peaks for catalyst nanomaterials.

		**Pd^0 $3d_{5/2}$**	**Pd^{2+} $3d_{5/2}$**	**Pd^0 $3d_{3/2}$**	**Pd^{2+} $3d_{3/2}$**
**$Pd/CeO_2/C$ NC**	**Position (eV)**	335.38	338.07	340.99	342.46
	**Area (%)**	37.35	16.92	35.93	9.79
**$Pd/CeO_2/C$ NS**	**Position (eV)**	335.99	337.59	340.63	343.57
	**Area (%)**	43.37	18.96	31.34	6.34
**$Pd/CeO_2/C$ NR**	**Position (eV)**	336.00	337.73	340.93	342.55
	**Area (%)**	51.60	20.58	24.19	3.63
**$Pd/CeO_2/C$ poly**	**Position (eV)**	335.43	337.02	340.73	342.78
	**Area (%)**	31.61	21.34	30.07	16.97
**Pd/C**	**Position (eV)**	335.42	337.26	341.06	342.99
	**Area (%)**	31.28	27.20	29.96	11.56
